# GATA1 repression-mediated ADRB2/cAMP/PKA signaling activation underlies the antidepressant effects of targeted geniposide delivery

**DOI:** 10.1038/s41420-026-03190-z

**Published:** 2026-06-11

**Authors:** Yu Chen, Yuetao Wen, Diru Xu, Lei Hao, Qinglin Liu, Jia Kuang, Wang Zhao, Zhao Yang

**Affiliations:** 1 Department of Neurology, Bishan Hospital of Chongqing Medical University, Chongqing, China; 2 Department of Neurosurgery, Chongqing University Jiangjin Hospital, Chongqing University, Chongqing, China; 3 Department of Dermatology, University-Town Hospital of Chongqing Medical University, Chongqing, China; 4 Department of Neurology, The Affiliated Yongchuan Hospital of Chongqing Medical University, Chongqing, China

**Keywords:** Drug discovery, Diseases

## Abstract

Depression is associated with complex hippocampal pathology, including disrupted signaling and neuronal damage. In this study, we developed a novel targeted nanoplatform using nucleic acid aptamer-modified astrocyte-derived extracellular vesicles loaded with geniposide (Apt-EVs@GP) to investigate its therapeutic mechanism, with a focus on the GATA binding protein 1 (GATA1)/adrenergic receptor beta 2 (ADRB2) axis in a chronic unpredictable mild stress (CUMS) mouse model. Apt-EVs@GP exhibited excellent biocompatibility and targeted accumulation in the hippocampus. Apt-EVs@GP treatment significantly ameliorated depression-like behaviors, as evidenced by enhanced locomotor activity and reduced immobility in standard behavioral assays. Transcriptomic analysis combined with molecular validation identified a key regulatory mechanism: Apt-EVs@GP suppressed stress-induced upregulation of GATA1, thereby relieving its transcriptional repression of ADRB2. This regulatory interaction was confirmed by chromatin immunoprecipitation and dual-luciferase reporter assays. Restoration of ADRB2 expression led to robust activation of the downstream cAMP/PKA/CREB signaling pathway. Functionally, reprogramming of the GATA1/ADRB2/cAMP axis conferred comprehensive hippocampal protection, including attenuation of neuroinflammation, suppression of neuronal apoptosis, and restoration of synaptic integrity. Our findings demonstrate that Apt-EVs@GP alleviates depression by targeting a novel GATA1/ADRB2 mechanism, highlighting its potential as a promising therapeutic strategy against depression-related hippocampal pathology.

**Figure:**
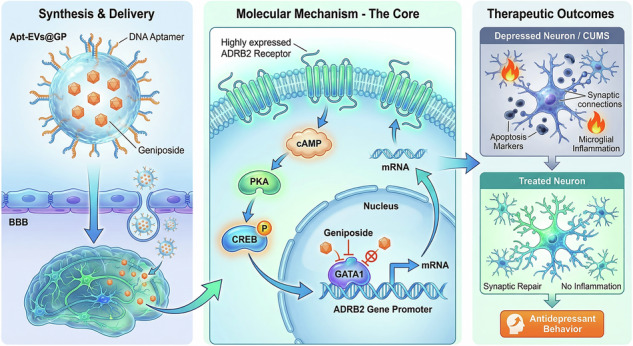

## Highlights

- This study reports the use of Apt-EVs@GP for the treatment of depression.
- Apt-EVs@GP improves depressive behavior by regulating the GATA1/ADRB2 signaling axis.
- Apt-EVs@GP alleviates neuroinflammation and restores synaptic integrity.
- Nucleic acid Apts enhance the targeting ability and stability of EVs.
- This study provides new therapeutic targets and theoretical insights for the treatment of depression.

## Introduction

Depression is a debilitating mental disorder with a steadily increasing global prevalence, imposing a substantial socioeconomic burden [1, 2]. Current pharmacological treatments, including selective serotonin reuptake inhibitors (SSRIs), are effective for a subset of patients. However, their clinical utility is limited by delayed therapeutic onset, treatment resistance in approximately 30% of individuals, and notable systemic side effects [3, 4]. These limitations underscore an urgent need for more precise therapeutic strategies that directly target the underlying neuropathological mechanisms of depression while minimizing off-target toxicity. In this context, the chronic unpredictable mild stress (CUMS) model has been widely employed as a robust preclinical platform. This model reliably recapitulates key depressive features, including neuroinflammation, neuronal apoptosis, and impairments in synaptic plasticity, thereby facilitating the evaluation of novel antidepressant interventions [5–7].

Geniposide (GP) is a bioactive iridoid glycoside derived from *Gardenia jasminoides* and is known for its pronounced neuroprotective and anti-inflammatory activities [8, 9]. Although GP has demonstrated antidepressant potential by crossing the blood-brain barrier (BBB) [10], its clinical translation is hindered by poor bioavailability and non-specific distribution. Extracellular vesicles (EVs) have emerged as promising endogenous nanocarriers due to their biocompatibility and intrinsic ability to traverse the BBB [11–13]. Moreover, surface functionalization of EVs with nucleic acid aptamers (Apts) can further enhance delivery precision by enabling active targeting and preferential accumulation at pathological sites [14]. Therefore, engineering an aptamer-functionalized EV system for GP delivery represents a rational strategy to overcome these limitations.

Mechanistically, the molecular etiology of depression remains incompletely understood. Accumulating evidence implicates dysregulation of the GATA binding protein 1 (GATA1)/β2-adrenergic receptor (ADRB2) signaling axis as a potential contributor to depressive pathogenesis. GATA1 is a stress-responsive transcription factor that has been shown to directly repress ADRB2 transcription [15, 16]. Given that ADRB2 signaling is crucial for neuroprotection and synaptic maintenance [17, 18], we hypothesized that stress-induced upregulation of GATA1 suppresses ADRB2, thereby exacerbating depressive pathology. Accordingly, therapeutic strategies aimed at restoring ADRB2 signaling by counteracting GATA1-mediated repression may represent a novel and mechanistically grounded approach for depression treatment.

In this study, we engineered a targeted nanoplatform consisting of aptamer-modified, astrocyte-derived EVs encapsulating geniposide (Apt-EVs@GP). This system was designed to exploit the high binding affinity of aptamers for the precise delivery of GP to the hippocampal microenvironment in mice subjected to CUMS. In this study, we integrated behavioral, transcriptomic, and molecular analyses to investigate the antidepressant effects of Apt-EVs@GP. We specifically examined whether Apt-EVs@GP alleviates depressive-like behaviors by modulating the GATA1/ADRB2 axis, activating the downstream cAMP/PKA/CREB signaling pathway, and mitigating neuroinflammation and synaptic damage. Collectively, this study provides both mechanistic insight and experimental evidence supporting a novel precision nanotherapeutic strategy for depression.

## Results

### Synthesis and characterization of Apt-EVs@GP

**Fig. 1:**
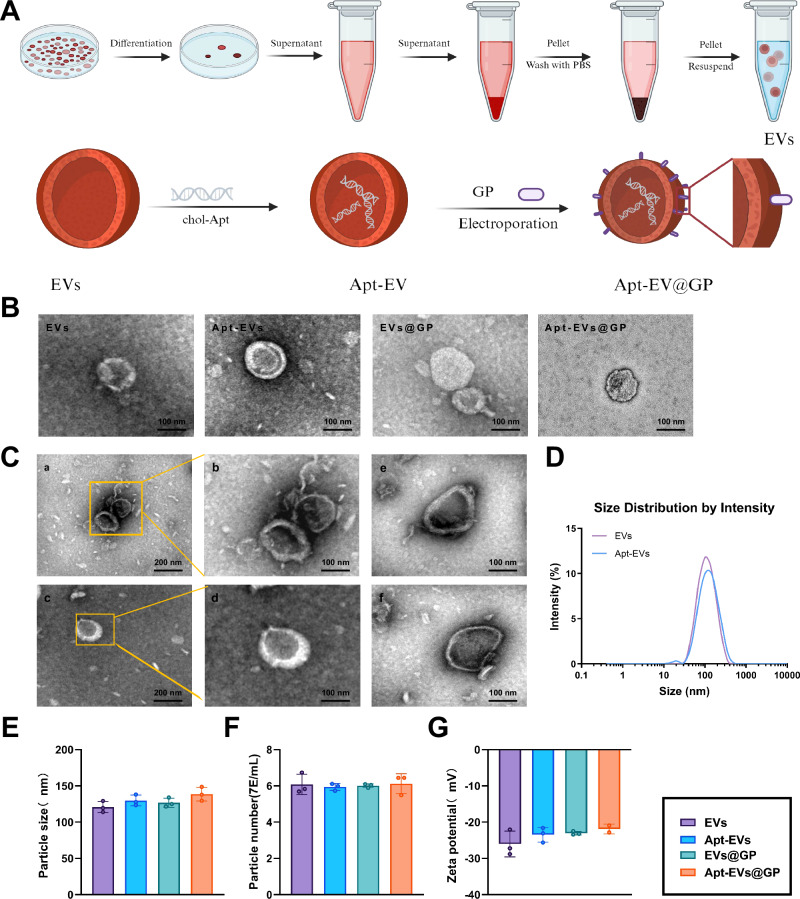
Synthesis, morphology, and characterization of Apt-EVs@GP. **A** Schematic diagram illustrating the synthesis process of Apt-EVs@GP. **B** Comparison of the morphology of EVs, Apt-EV, EV@GP, and Apt-EVs@GP as observed by Cryo-TEM (scale bar: 100 nm); **C** Morphological comparison of EVs, Apt-EV, EV@GP, and Apt-EVs@GP by TEM. **D** Particle size distribution of EVs and Apt-EV measured by DLS. **E** Particle size distribution of EVs, Apt-EV, EV@GP, and Apt-EVs@GP measured by NTA. **F** Particle concentration (particles per milliliter) of EVs, Apt-EV, EV@GP, and Apt-EVs@GP. **G** Zeta potential measurements of EVs, Apt-EV, EV@GP, and Apt-EVs@GP.

Following the established protocol, EVs were isolated from astrocytes through ultracentrifugation of their conditioned medium. The EVs were then modified with nucleic acid Apts and loaded with GP (Fig. 1A). Comprehensive characterization of EVs, Apt-EVs, and Apt-EVs@GP was performed using TEM, NTA, zeta potential measurement, GPyo-TEM, UV-Vis-NIR absorption spectroscopy, and WB.

TEM and Cryo-TEM imaging demonstrated that EVs displayed a typical cup-shaped morphology (Fig. 1B, C), similar to Apt-EVs. DLS measurements (Fig. 1D) showed that EVs were uniformly distributed with a mean size of 104.3 ± 1.8 nm. Following aptamer modification, particle size increased significantly, with a further enlargement observed after drug loading. NTA showed that native EVs had an average diameter of 116.3 ± 1.3 nm (Fig. 1E), whereas Apt-EVs exhibited a modest increase in size. Notably, no significant difference in particle concentration was detected between EVs and Apt-EVs (Fig. 1F).

Zeta potential measurements revealed that the surface ζ-potential of EVs was −24.8 mV, whereas the ζ-potential of Apt-EVs increased to −21.6 mV (Fig. 1G), confirming the successful conjugation of the Apts. Apt-EVs@GP showed similar morphology, ζ-potential, and nanoparticle count per milliliter to EV@GP, with the only noticeable difference being a slightly larger average diameter (Fig. 1C–G). Notably, compared to Apt-EV, Apt-EVs@GP exhibited favorable consistency across all parameters.

To investigate whether EVs altered the intrinsic properties of GP, we examined the photophysical properties of Apt-EVs@GP in solution. UV-Vis-NIR absorption spectra (Fig. S1A) showed that the absorption profiles of free GP and Apt-EVs@GP were similar, with both exhibiting an absorption peak around 280 nm. These results indicate that EV encapsulation did not affect the optical properties or appearance of GP, suggesting preservation of its inherent physicochemical characteristics.

Furthermore, total internal reflection fluorescence microscopy (TIRFM) demonstrated strong spatial colocalization between FAM-labeled aptamers and the red fluorescent signal from the EV membrane (Fig. S1B), confirming the successful modification of the Apt on the EV membrane. Co-incubation of EVs with cholesterol-conjugated and unconjugated Apts demonstrated that only the cholesterol-modified group retained FAM fluorescence after ultrafiltration, verifying cholesterol-mediated incorporation (Fig. S2A).

As shown in Fig. S1C, the LC of EVs reached a maximum of 8.4 ± 0.3 μg GP per 10^10^ EVs, corresponding to an EE of 31.8 ± 0.6% within 3 h. Prolonging the loading duration to 4 h did not yield a further significant increase, indicating saturation of GP loading. This process is primarily driven by the transmembrane pH gradient, which promotes the diffusion of non-ionized GP from the external medium into the vesicular lumen. Quantitative fluorescence calibration enabled estimation of the number of Apt chains incorporated per EV, revealing a positive correlation between Apt density and the initial concentration of cholesterol-conjugated Apt (Fig. S1D). WB analysis further verified the successful isolation of astrocyte-derived EVs, as evidenced by the enrichment of canonical EV markers (CD9, CD63, CD81, Alix, and TSG101) and the absence of the endoplasmic reticulum marker Calnexin. Importantly, Apt functionalization did not alter the expression of EV marker proteins (Fig. S1E).

PKH67-labeled EVs demonstrated a time-dependent increase in astrocytic uptake (Fig. S1F–G), confirming efficient internalization of Apt-EVs. These results indicate that cholesterol-mediated modification enables stable and dense Apt anchoring on the EV surface, providing a robust and straightforward strategy for constructing functional Apt-EV nanostructures.

### Stability and drug release of Apt-EVs@GP

**Fig. 2:**
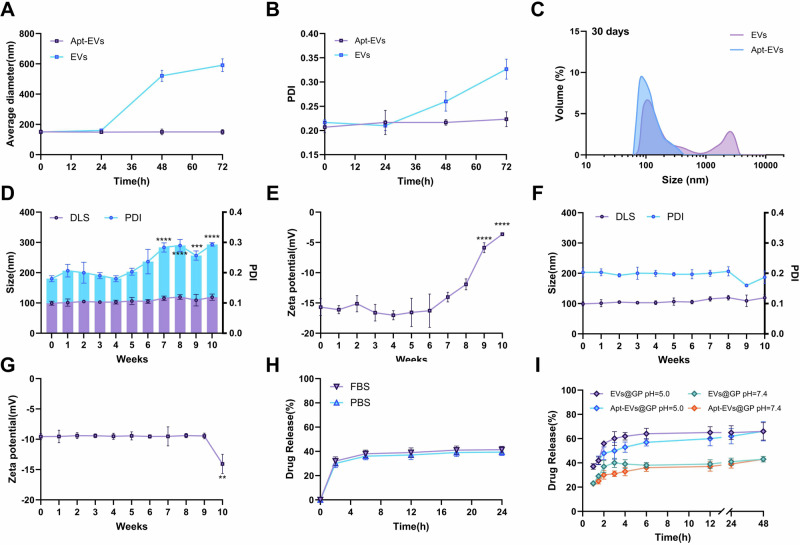
Stability and drug release characteristics of native EVs and Apt-EVs. **A** Average particle size changes of native EVs and Apt-EVs after 3 days of storage at room temperature. **B** Changes in the PDI after 3 days of storage at room temperature. **C** Size distribution of EVs and Apt-EVs after 30 days of storage at room temperature. **D** Changes in particle size of unmodified EVs stored at 4 °C, measured by DLS. **E** Changes in the Zeta potential of unmodified EVs stored at 4 °C. **F** Changes in particle size of Apt-EVs stored at 4 °C, measured by DLS. **G** Changes in the Zeta potential of Apt-EVs stored at 4 °C. **H** Release curves of GP from Apt-EVs@GP in PBS and FBS buffers. **I** Cumulative release curves of GP from EVs or Apt-EVs under normal physiological (PBS, pH 7.4) or acidic (acetate buffer, pH 5.0) conditions (mean ± standard deviation (SD), with three experimental replicates). *p < 0.05, **p < 0.01, ***p < 0.001, ****p < 0.0001.

To evaluate Apt-EVs’ stability, both natural EVs and Apt-EVs were stored in PBS. DLS data showed that, after 3 days of storage, natural EVs’ average particle size and PDI increased (Fig. 2A, B; Fig. S2D, E), indicating potential vesicle rupture or fusion. TEM images also revealed the presence of large, polydisperse structures (Fig. S2C). In contrast, the size distribution of Apt-EVs remained consistent after 30 days (Fig. 2C; Fig. S2D), demonstrating improved stability. Figure 2D, E shows that, after 7 weeks, unmodified EVs exhibited significant increases in particle size and PDI, with the zeta potential approaching 0 mV, suggesting fusion and aggregation. However, Apt-EVs remained stable for up to 9 weeks, with zeta potential values gradually shifting from −15 mV to −20 mV (Fig. 2F, G), likely due to the protective effect of the nucleic acid chain.

To assess the stability of GP in serum, Apt-EVs@GP, EV@GP, and free GP were incubated with FBS. The results showed that free GP degraded within 6 h, while GP encapsulated in EVs remained detectable after 12 h (Fig. S2B). Release experiments indicated that Apt-EVs@GP released approximately 9% of the drug within 2 h and 93% after 24 h (Fig. 2H). Both EV@GP and Apt-EVs@GP exhibited enhanced drug release under acidic pH conditions, with Apt modification slightly delaying GP release (Fig. 2I). These results suggest that Apt-EVs possess favorable stability and sustained drug-release properties, making them suitable for targeted delivery applications.

### In vitro targeting and biocompatibility of Apt-EVs@GP

**Fig. 3:**
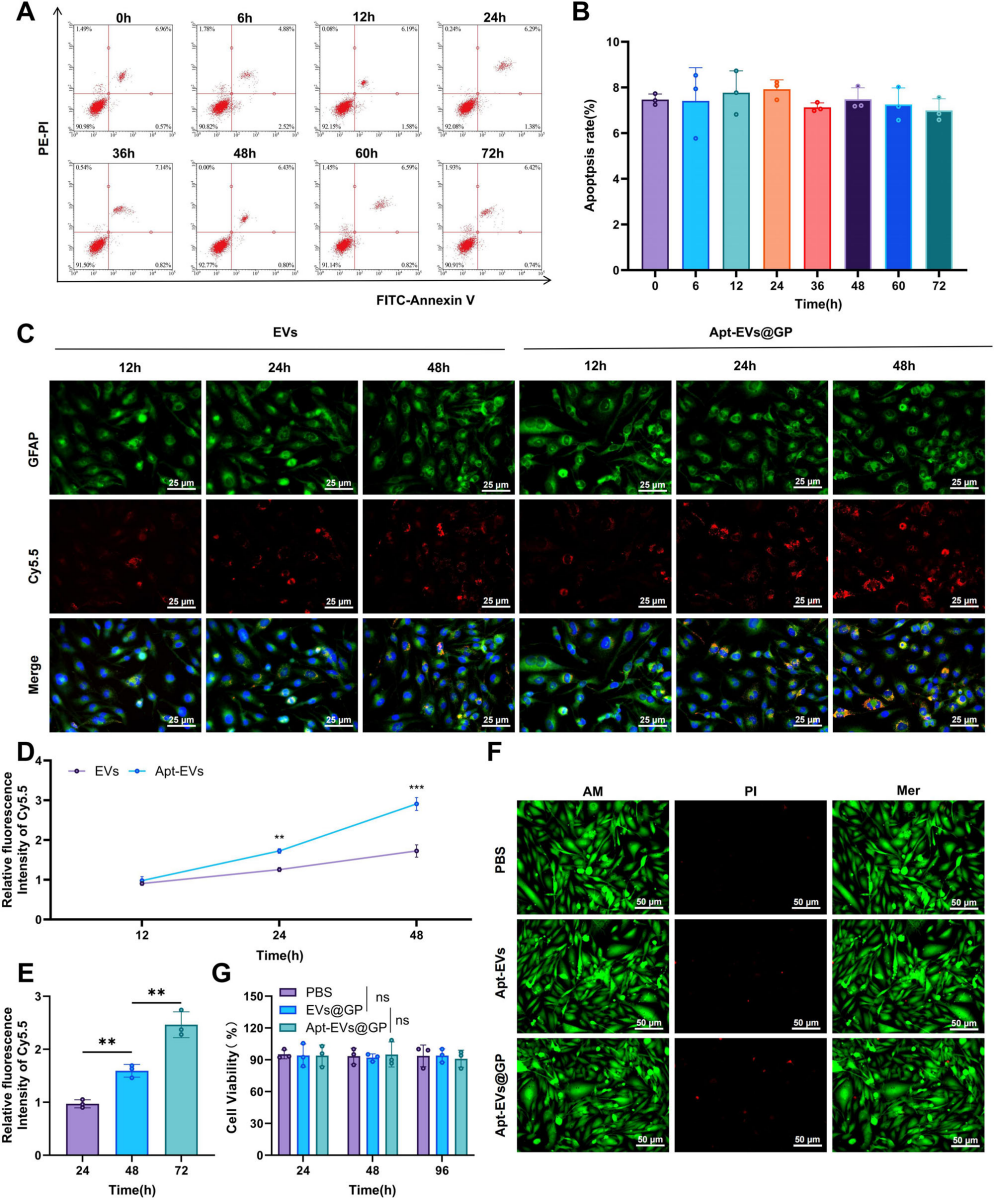
Effects of Apt-EVs@GP on cell apoptosis, uptake efficiency, and cytotoxicity. **A** Flow cytometry analysis assessing the apoptosis effects of Apt-EVs@GP at different time points. **B** Data presented as mean ± SEM for (**A**). **C** Confocal microscopy images of astrocytes following TGF-β treatment at different time points, showing the uptake of Apt-EV and EVs (scale bar: 25 μm). **D** Uptake efficiency of Apt-EVs and EVs in astrocytes after 72 h of TGF-β treatment. **E** Changes in uptake efficiency of Apt-EVs by astrocytes over time following TGF-β treatment. **F** Confocal microscopy images showing cytotoxicity of PBS, Apt-EV, and Apt-EVs@GP in astrocytes, using Calcein-AM and PI staining. **G** CCK-8 assay evaluating the effect of Apt-EVs@GP on cell viability. *p < 0.05, **p < 0.01, ***p < 0.001.

FCM analysis showed that Apt-EVs@GP did not induce significant apoptosis in astrocytes at different time points (Fig. 3A, B), indicating negligible cytotoxicity. Given the critical role of neurons in information transmission and integration within the nervous system, potential neurotoxicity was further evaluated using NeuN immunofluorescence/TUNEL double staining. The results revealed no detectable neuronal apoptosis following Apt-EVs@GP treatment (Fig. S3A, B). Moreover, immunofluorescence labeling confirmed that Apt-EVs exhibited neuronal targeting (Fig. S3C). Together, these findings indicate that engineered EVs maintain biocompatibility comparable to natural EVs, and Apt-EVs can target neuronal cells.

Chondroitin sulfate proteoglycans (CSPGs) are well-established inhibitors of axonal regeneration and are markedly upregulated in activated astrocytes [19, 20]. TGF-β treatment was used to induce CSPG expression in astrocytes, thereby simulating an activated state [20]. The targeting efficiency of Apt-EVs was then assessed by confocal microscopy. Compared with EVs, Apt-EVs exhibited significantly greater uptake in TGF-β-treated astrocytes, with fluorescence intensity increasing in a time-dependent manner (Fig. 3C). After 72 h of TGF-β stimulation, uptake efficiency of Apt-EVs was markedly higher than that of EVs (Fig. 3D). Similarly, Apt-EVs@GP showed progressively enhanced uptake with prolonged TGF-β exposure (Fig. 3E). Collectively, these results demonstrate that Apt-EVs selectively recognize and are efficiently internalized by CSPG-expressing astrocytes, confirming that aptamer functionalization substantially enhances the targeting affinity and specificity of EVs.

To assess the cytotoxicity of Apt-EVs@GP, astrocytes were stained with Calcein-AM and PI and examined using confocal microscopy (Fig. 3F). In the PBS, Apt-EVs, and Apt-EVs@GP groups without irradiation, the majority of cells displayed strong Calcein-AM fluorescence with minimal PI staining, indicating negligible dark cytotoxicity. Consistently, CCK-8 assays showed no significant reduction in cell viability following Apt-EVs@GP treatment (Fig. 3G). These findings suggest that Apt-EVs@GP exhibit favorable in vitro biocompatibility.

### Apt-EVs@GP exhibit enhanced targeting ability and biocompatibility in vitro

**Fig. 4:**
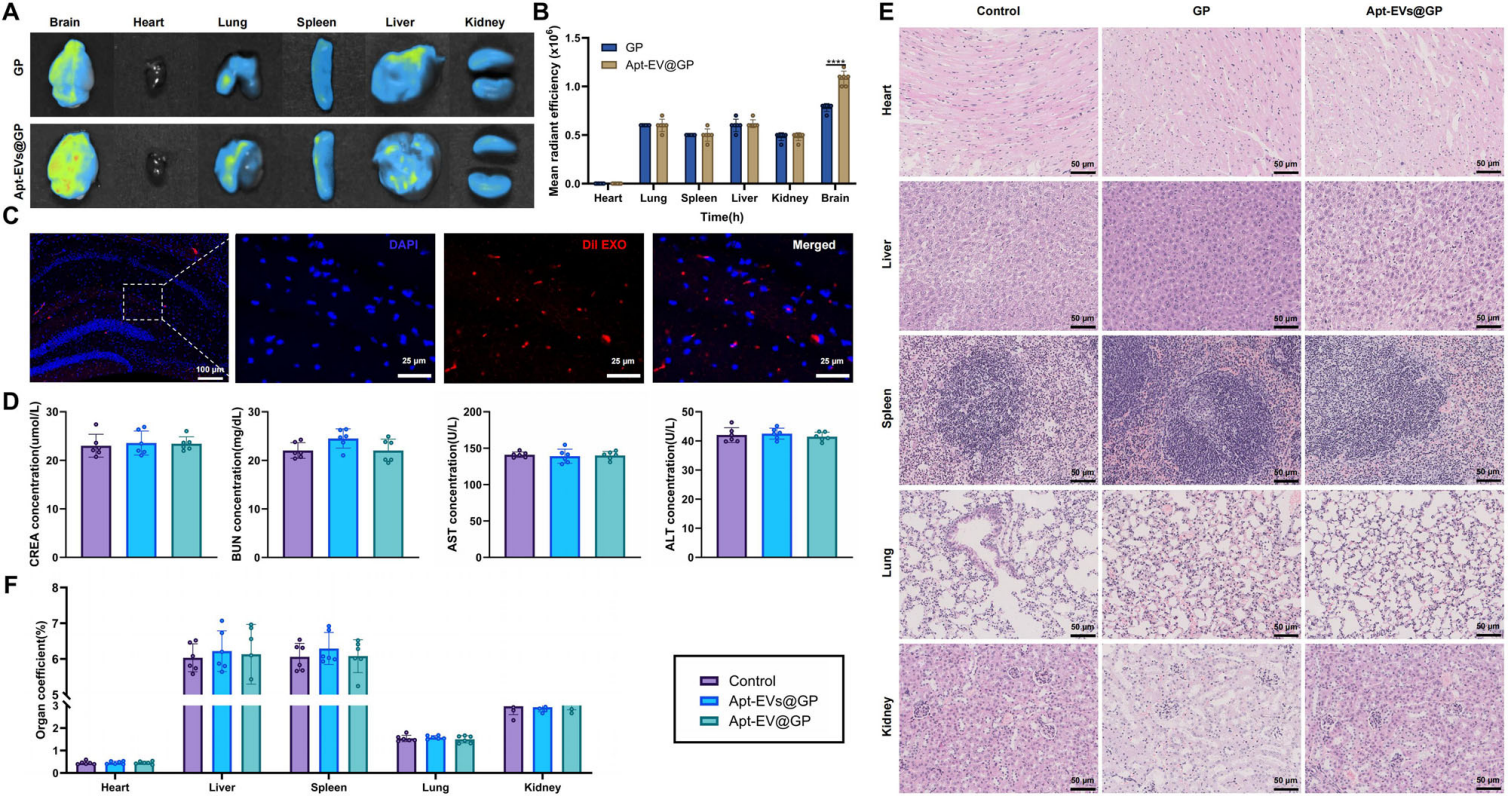
In vivo targeting and biocompatibility assessment of Apt-EVs@GP. **A** IVIS showing the distribution of EVs in mouse tissues. **B** Quantification of fluorescence intensity in different tissues of each mouse group. **C** Fluorescence imaging of tissue sections showing accumulation of EVs in target tissues. **D** Serum biochemical analysis (ALT, AST, BUN, CREA) for each group of mice. **E** H&E staining to observe structural changes in mouse tissues. **F** Organ-to-body weight ratios for different organs. Data are expressed as mean ± standard error (SE). *p < 0.05, **p < 0.01, ***p < 0.001, ns indicates no significant difference. *N* = 6.

IVIS revealed that Apt-EVs@GP preferentially accumulated in the brain, whereas free GP displayed widespread systemic distribution. Quantitative fluorescence analysis revealed significantly higher signal intensity in the brain compared with other major organs, confirming the enhanced targeting capability conferred by vesicle modification (Fig. 4A, B). Further fluorescence imaging of tissue sections showed that Apt-EVs@GP was mainly localized in the hippocampus (Fig. 4C). To assess biosafety, serum biochemical markers (ALT, AST, BUN, CREA) in the Apt-EVs@GP group remained within normal ranges and showed no significant differences compared with the control group (Fig. 4D). Furthermore, histopathological examination of major organs (heart, liver, spleen, lung, and kidney) by H&E staining revealed no detectable tissue damage or inflammatory lesions in the Apt-EVs@GP–treated group (Fig. 4E, F). These results collectively demonstrate that Apt-EVs@GP possess enhanced targeting ability and biocompatibility.

### Apt-EVs@GP improve depressive-like symptoms in mice

**Fig. 5:**
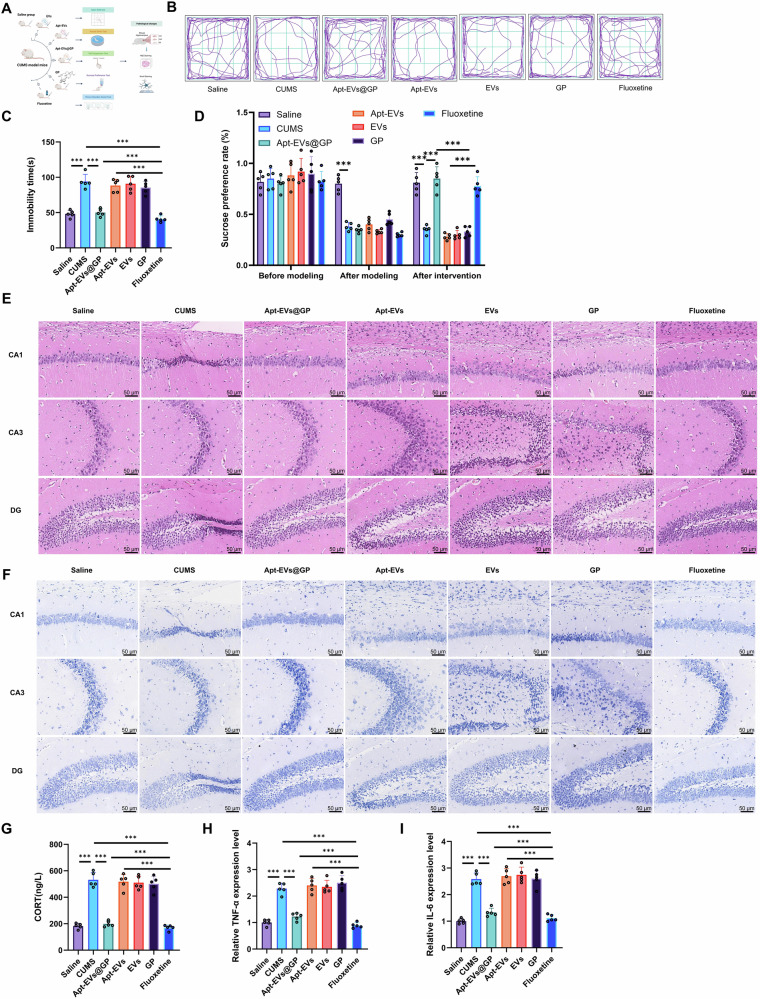
Apt-EVs@GP alleviates depressive-like symptoms in mice. **A** Experimental design and grouping. **B** Open field test. **C** Forced swim and tail suspension tests. **D** Sucrose preference test. **E** H&E staining of hippocampal CA1, CA3, and DG regions. **F** Nissl staining of hippocampal CA1, CA3, and DG regions. **G** Serum corticosterone levels. **H**, **I** Serum TNF-α and IL-6 levels. Data are expressed as mean ± SEM. *p < 0.05, **p < 0.01, ***p < 0.001, ns not significant. *N* = 6.

**Fig. 6:**
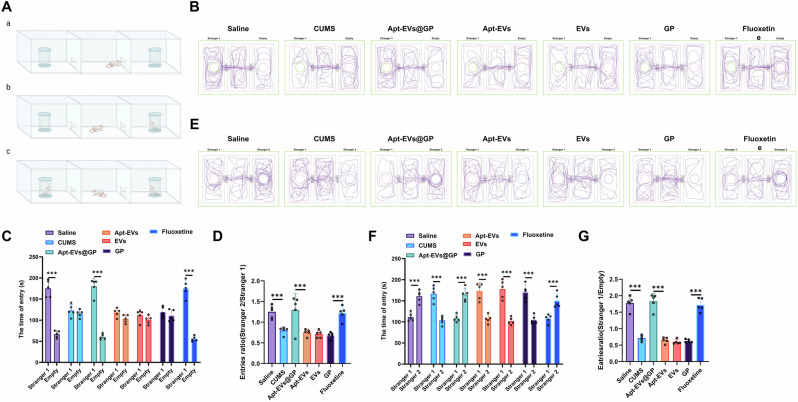
Three-chamber social interaction test (ST). **A** Schematic of ST protocols: a, habituation phase; b, phase I; c, phase II. **B** Locomotor traces of mice during phase I. **C**, **D** Time spent in Stranger 1 vs. empty cage during phase I. **E** Locomotor traces during phase II. **F**, **G** Time spent with Stranger 1 vs. Stranger 2 and empty cage during phase II. Data are expressed as mean ± SEM. *p < 0.05, **p < 0.01, ***p < 0.001, ns not significant. *N* = 6.

To evaluate the antidepressant efficacy of Apt-EVs@GP, CUMS mice were treated with EVs, Apt-EVs, Apt-EVs@GP, GP alone, or fluoxetine, followed by comprehensive behavioral and inflammatory assessments. The experimental design and treatment regimen are summarized in Fig. 5A. Compared with control mice, CUMS-exposed mice exhibited pronounced depression-like phenotypes, including reduced locomotor activity in the OFT, prolonged immobility in the forced swim and tail suspension tests, diminished sucrose preference, and impaired social interaction in the three-chamber test (lack of preference for Stranger 1 in phase I or Stranger 2 in phase II). These findings confirmed the robust establishment of the CUMS model. Treatment with Apt-EVs@GP or fluoxetine significantly improved these behavioral deficits, whereas EVs, Apt-EVs, and GP alone showed weaker effects (Fig. 5B–D, Fig. 6).

Histological examination using H&E and Nissl staining revealed severe neuronal damage in the hippocampal CA1, CA3, and DG regions of CUMS mice, including neuronal loss, loose arrangement, nuclear pyknosis, atrophy, and reduced Nissl bodies. Apt-EVs@GP or fluoxetine treatment markedly ameliorated these pathological changes, showing restored neuronal arrangement and increased Nissl bodies, whereas EVs, Apt-EVs, and GP groups showed no significant recovery (Fig. 5E, F).

Serum corticosterone levels were markedly elevated in CUMS mice but were significantly reduced following treatment with Apt-EVs@GP or fluoxetine, whereas EVs, Apt-EVs, and GP alone produced only partial recovery (Fig. 5G). Similarly, proinflammatory cytokines TNF-α and IL-6 were significantly reduced in Apt-EVs@GP and fluoxetine groups, while EVs, Apt-EVs, and GP groups showed increased levels (Fig. 5H, I). Collectively, these results indicate that Apt-EVs@GP, similar to fluoxetine, significantly alleviated depressive-like behaviors, restored neuronal injury, reduced corticosterone levels, and suppressed proinflammatory cytokine secretion, whereas single-agent treatments were less effective.

### Apt-EVs@GP modulates ADRB2 expression by inhibiting GATA1 upregulation

**Fig. 7:**
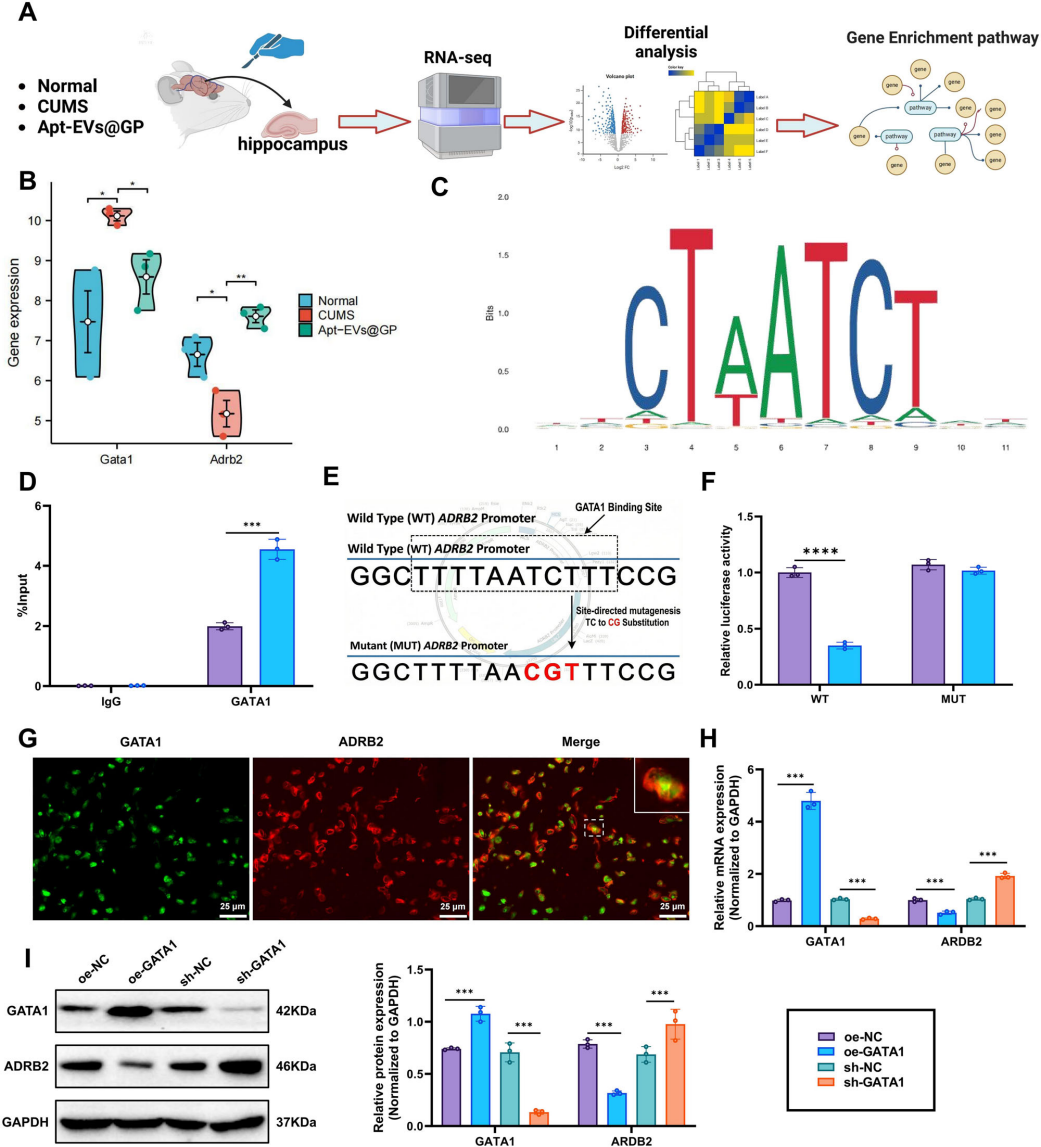
Apt-EVs@GP inhibits GATA1 to upregulate ADRB2 expression. **A** Experimental workflow, illustrating the RNA sequencing and analysis process for hippocampal tissue from normal, CUMS model, and Apt-EVs@GP treatment groups. **B** Expression changes of GATA1 and ADRB2 in the normal, CUMS, and Apt-EVs@GP treatment groups. **C**, **D** ChIP analysis showing GATA1 binding to the ADRB2 promoter region. **E** Schematic representation of the Wild-Type (WT) ADRB2 promoter sequence and the Mutant (MUT) sequence containing base substitutions in the predicted GATA1 binding site. **F** Dual luciferase reporter assay showing relative luciferase activity in HEK293T cells co-transfected with GATA1 and either WT or MUT ADRB2 promoter plasmids. The repression observed in the WT group was abolished in the MUT group. **G** IF staining showing co-localization of GATA1 and ADRB2 in tissue. **H**, I RT-qPCR and Western blot analysis of mRNA and protein expression levels of ADRB2 under conditions of GATA1 overexpression or knockdown. All data are presented as mean ± SEM. Experiments were repeated three times, and statistical analysis was performed using ANOVA followed by Tukey’s post hoc test. *p < 0.05, **p < 0.01, ***p < 0.001, ****p < 0.0001.

To elucidate the molecular mechanisms underlying the antidepressant effects of Apt-EVs@GP, we performed transcriptome sequencing of hippocampal tissue from normal mice, CUMS model mice, and Apt-EVs@GP-treated mice, and systematically analyzed the gene expression differences across the groups (Fig. 7A). Differential expression analysis revealed substantial transcriptional alterations between CUMS and normal mice, as well as between Apt-EVs@GP–treated and untreated CUMS mice (Fig. S4A, B). Intersection analysis further identified 146 overlapping differentially expressed genes (Fig. S4C) that were concurrently dysregulated in the CUMS condition and reversed upon Apt-EVs@GP treatment. To gain functional insight into this gene set, pathway enrichment analyses were subsequently performed.

KEGG pathway enrichment analysis (Fig. S4D) demonstrated that the overlapping genes were significantly enriched in pathways including Neuroactive ligand-receptor interaction, Cytokine-cytokine receptor interaction, and the Apelin signaling pathway. These results suggest that these genes are closely associated with the regulation of neurotransmission and immune responses. Consistently, GO functional enrichment analysis (Fig. S4E) further revealed that these genes play crucial roles in “sulfur transferase activity,” “receptor-ligand activity,” and “secondary active transporter activity,” indicating their potential role in regulating the extracellular matrix, signal transduction, and cell adhesion.

PCA revealed a clear separation between CUMS model mice and normal controls, indicating that depressive-like states are associated with pronounced transcriptional alterations (Fig. S5A). Notably, the gene expression profile of Apt-EVs@GP–treated mice was distinct from that of untreated CUMS mice and clustered closer to the normal group, suggesting that Apt-EVs@GP partially restored CUMS-induced transcriptional dysregulation. Intersection analysis further identified 55 key genes that were significantly upregulated in the CUMS versus normal comparison and concurrently downregulated following Apt-EVs@GP treatment (Fig. S5B, C). Conversely, eight genes that were downregulated in CUMS mice and upregulated after Apt-EVs@GP administration exhibited expression levels approaching those of normal controls (Fig. S5D, E). Together, these findings indicate that Apt-EVs@GP reverses a subset of CUMS-associated gene expression changes toward a normal transcriptional state.

Further analysis of key genes identified GATA1 as the most significantly altered transcription factor. As an upregulated transcription factor, GATA1 may affect the depression model by regulating downstream genes [21]. In addition, ADRB2, a key receptor regulated by neurotransmitters, showed significantly reduced expression in the CUMS group, suggesting that depression may influence mood regulation by inhibiting ADRB2 signaling [22] (Fig. 7B). We hypothesize that treatment with Apt-EVs@GP may relieve the suppression of ADRB2 expression by inhibiting the upregulation of GATA1, thereby promoting the restoration of ADRB2.

ChIP experiments further demonstrated that GATA1 was significantly enriched in the ADRB2 promoter region (Fig. 7C, D). To precisely identify the binding site, we performed site-directed mutagenesis assays. We constructed a mutant ADRB2 promoter reporter (MUT) by altering the core GATA1 binding motif from TTTTAATCTTT to TTTTAACGTTT (Fig. 7E). Dual-luciferase reporter assays showed that GATA1 overexpression markedly suppressed the transcriptional activity of the WT ADRB2 promoter, this inhibitory effect was completely abolished in the MUT group (Fig. 7F). These results indicate that GATA1 represses ADRB2 transcription through direct binding to the TTTTAATCTTT motif. Consistently, IF analysis revealed pronounced co-localization of GATA1 and ADRB2 within the same cellular regions (Fig. 7G), supporting the hypothesis that GATA1 regulates ADRB2 expression.

RT-qPCR and WB analyses demonstrated that GATA1 overexpression decreased ADRB2 mRNA and protein levels, whereas silencing GATA1 led to a marked increase in ADRB2 expression (Fig. 7H-I). These results collectively suggest that GATA1 directly inhibits ADRB2 expression by binding to its promoter region.

### Apt-EVs@GP Alleviates Depressive-like Behavior and Reduces Inflammation in CUMS Mice *via* the GATA1/ADRB2 Axis

**Fig. 8:**
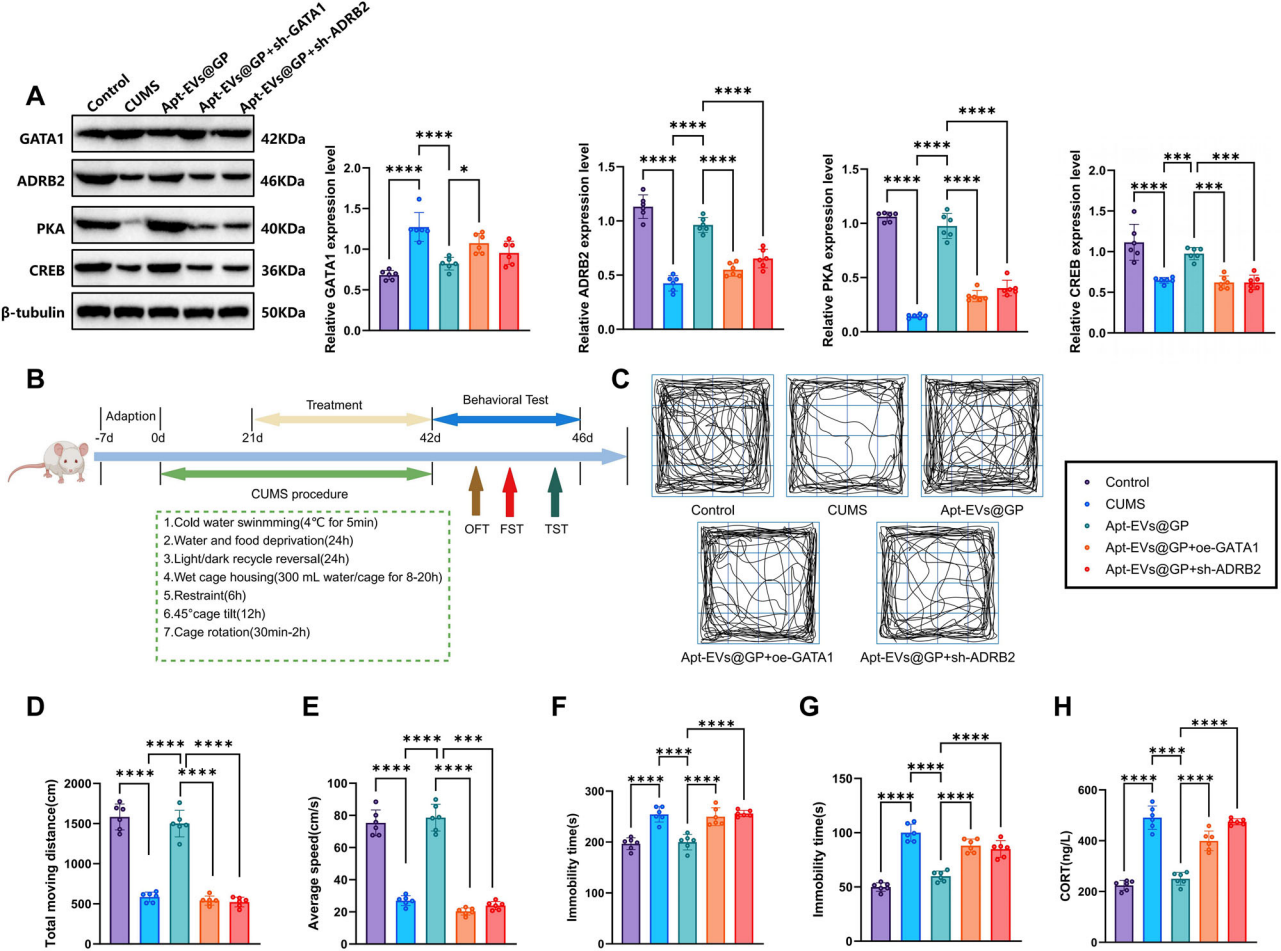
Effects of Apt-EVs@GP on depressive-like behavior and inflammatory response in CUMS mice *via* the GATA1/ADRB2 signaling axis. **A** Western blot results show changes in GATA1, ADRB2, PKA, and CREB protein levels in brain tissues of CUMS mice after the indicated treatments. **B** Schematic timeline of the experiment. **C** Pathways of movement for mice in each group during the OFT. **D**, **E** Total distance traveled by mice in each group during the OFT. **E** Average speed of mice in each group during the OFT. **F** Immobility time in the forced swimming test for each group of mice. **G** Immobility time in the TST for each group of mice. **H** Serum cortisol levels in mice from each group. Data are presented as mean ± SEM. *p < 0.05, **p < 0.01, ***p < 0.001, ****p < 0.0001. *N* = 6.

As shown in Fig. S6, the experimental workflow for in vivo studies in the Apt-EVs@GP treatment group is outlined. qRT-PCR analysis showed that GATA1 mRNA levels were significantly upregulated in CUMS mice compared with controls, whereas ADRB2 mRNA expression was markedly reduced (Fig. S7A, B). These transcriptional changes were further validated at the protein level by WB analysis, which demonstrated increased GATA1 and decreased ADRB2 expression in the brains of CUMS mice (Fig. 8A). These results indicate that CUMS-induced depressive-like behaviors are accompanied by increased GATA1 expression and decreased ADRB2 expression.

In the treatment group, Apt-EVs@GP increased the mRNA and protein levels of ADRB2 and reduced GATA1 expression (Fig. 8A). In contrast, the Apt-EVs@GP + oe-GATA1 group exhibited a significant increase in GATA1 expression and a corresponding decrease in ADRB2 expression, while the Apt-EVs@GP + sh-ADRB2 group demonstrated a notable reduction in ADRB2 levels compared with the Apt-EVs@GP group. Consistently, the levels of downstream signaling components, including cAMP, PKA, and CREB, were reduced in both groups (Fig. 8A; Figure S7C–E). These findings suggest that Apt-EVs@GP modulates the expression of GATA1 and ADRB2, thereby activating downstream signaling pathways to improve the depressive-like behaviors induced by CUMS.

Behavioral studies further corroborated these findings. The experimental timeline is illustrated in Fig. 8B. CUMS model mice exhibited typical depressive-like behaviors, characterized by reduced total movement distance and average speed in the OFT, as well as increased immobility time in the FST and TST. Treatment with Apt-EVs@GP significantly improved these behaviors, as evidenced by increased movement distance and speed in the OFT (Fig. 8C–E) and reduced immobility times in both the FST and TST (Fig. 8F, G). In contrast, the improvement in behavior was less pronounced in the Apt-EVs@GP + oe-GATA1 and Apt-EVs@GP + sh-ADRB2 groups, highlighting the crucial roles of GATA1 and ADRB2 in behavioral recovery. Specifically, in the Apt-EVs@GP + sh-ADRB2 group, the therapeutic effects were significantly blocked, with mice exhibiting prolonged immobility times comparable to the CUMS model, confirming that functional ADRB2 is required for the antidepressant efficacy (Fig. 8F, G).

Further analysis showed a significant increase in serum cortisol levels in the CUMS model group. In the Apt-EVs@GP group, cortisol levels were markedly reduced, while cortisol levels rebounded in the Apt-EVs@GP + oe-GATA1 and Apt-EVs@GP + sh-ADRB2 groups (Fig. 8H). Notably, the rebound in cortisol levels in the sh-ADRB2 group further validated that the stress-response modulation is dependent on the ADRB2 receptor. The expression levels of inflammatory cytokines TNF-α and IL-6 were significantly lower in the Apt-EVs@GP group, but these levels increased again in the Apt-EVs@GP + oe-GATA1 and Apt-EVs@GP + sh-ADRB2 groups (Fig. S7F–H).

Taken together, these results indicate that Apt-EVs@GP significantly improves depressive-like behavior in CUMS model mice and effectively reduces inflammation by upregulating the expression of GATA1 and ADRB2, thereby activating downstream signaling pathways.

### Apt-EVs@GP Alleviates Neuroinflammation, Cell Apoptosis, and Synaptic Damage in the CUMS Mouse Model by Modulating the GATA1/ADRB2 Signaling Axis

Histological analyses using H&E and Nissl staining revealed marked pathological differences in the hippocampal regions among the experimental groups. In control mice, neurons within the CA1, CA3, and DG regions were abundant, densely arranged, and morphologically intact, with well-defined cell membranes and uniform cytoplasmic staining. In contrast, CUMS model mice exhibited a pronounced reduction in neuronal density, accompanied by loose cellular organization, nuclear condensation, cell shrinkage, and irregular morphology, indicating severe neuronal damage (Fig. S8A). Consistent with these observations, Nissl staining showed densely packed neurons with abundant Nissl bodies in control mice, whereas CUMS mice displayed sparse neuronal distribution, shrunken cell bodies, and a marked loss of Nissl substance, suggesting impaired protein synthesis capacity (Fig. S8B). Treatment with Apt-EVs@GP substantially ameliorated these histopathological abnormalities, as evidenced by restored neuronal organization, improved cytoplasmic morphology, and increased Nissl body content, indicating partial recovery of neuronal integrity and protein synthesis. However, this structural restoration was abolished in the Apt-EVs@GP + sh-ADRB2 group. Histological analysis of CA1, CA3, and DG regions revealed a recurrence of neuronal shrinkage, loose arrangement, and nuclear pyknosis, similar to the CUMS group (Fig. S8A).

IF staining revealed an increase in Iba-1 positive microglia in the CUMS model group, suggesting enhanced microglial activation (Fig. S8C, D). Additionally, GFAP staining showed a significant proliferation of astrocytes in the CUMS group (Fig. S8E, F). Following Apt-EVs@GP treatment, the number of Iba-1-positive microglia and the proliferation of astrocytes were markedly reduced, indicating improvement in neuroinflammation. Importantly, ADRB2 knockdown abolished these protective effects, leading to a re-emergence of activated, amoeboid microglia, as evidenced by increased Iba-1/DAPI co-localization and a significant rise in microglial cell numbers compared with the Apt-EVs@GP–treated group (Fig. S8C, D). Moreover, relative to CUMS mice, Apt-EVs@GP treatment increased neuronal density in the CA3 and DG regions and markedly improved neuronal organization, further supporting its neuroprotective role.

TUNEL staining further validated the anti-apoptotic effects of Apt-EVs@GP. In the CUMS model group, a substantial increase in TUNEL-positive neurons was observed in the hippocampus, indicating pronounced apoptosis. In contrast, the Apt-EVs@GP treatment group exhibited a marked reduction in apoptotic cells, highlighting its neuroprotective role in suppressing apoptosis and preserving neuronal integrity (Fig. S8G, H). This anti-apoptotic effect was dependent on ADRB2, as the sh-ADRB2 group showed a high density of TUNEL-positive cells (Green) colocalized with DAPI (Blue), and the percentage of apoptotic cells rebounded to levels comparable to the CUMS model (Fig. S8G, H).

Electron microscopy analysis revealed a decrease in synaptic density and significant synaptic damage in the CUMS model group. After Apt-EVs@GP treatment, synaptic density was significantly restored, and the integrity of the synaptic structure was markedly improved (Fig. S9A–G). However, this restorative effect was significantly inhibited in the oe-GATA1 and sh-ADRB2 treatment groups (Fig. S8A–H; Fig. S9A–G).

In conclusion, Apt-EVs@GP modulates the GATA1/ADRB2 signaling axis, significantly improving neuronal damage, neuroinflammation, apoptosis, and synaptic damage in the CUMS mouse model. The reversal of these benefits upon ADRB2 knockdown confirms the essential role of this receptor in the therapeutic mechanism.

## Discussion

Current antidepressant therapies are often constrained by delayed therapeutic onset, treatment resistance, and systemic adverse effects, underscoring the urgent need for more precise and targeted interventions [23, 24]. In this study, we sought to overcome these limitations by developing a novel nanotherapeutic platform, Apt-EVs@GP, and systematically validating its efficacy in a CUMS mouse model. In contrast to conventional pharmacological approaches that depend on nonspecific systemic distribution, our strategy exploits the intrinsic BBB permeability of EVs combined with aptamer-mediated targeting to deliver GP directly to the hippocampal microenvironment. Moreover, by identifying the GATA1/ADRB2 signaling axis as a critical molecular target, our findings not only demonstrate the robust antidepressant efficacy of Apt-EVs@GP but also elucidate a mechanistic framework that distinguishes this targeted nanotherapy from existing antidepressant treatments.

A central innovation of this study is the development of the Apt-EVs@GP delivery system. While GP possesses potent neuroprotective properties, its clinical utility is hindered by poor bioavailability and limited BBB permeability [25]. Consistent with these limitations, the modest therapeutic effects observed for free GP and unmodified EVs in our study are likely attributable to insufficient accumulation within the hippocampal lesion microenvironment. By integrating the intrinsic biocompatibility and BBB permeability of astrocyte-derived EVs with the active targeting capability of nucleic acid aptamers, Apt-EVs@GP achieved precise and efficient hippocampal localization. This targeted delivery strategy substantially enhanced the antidepressant efficacy of GP, enabling robust behavioral improvement and tissue repair at reduced systemic doses compared with conventional treatment regimens [26, 27]. The specificity of this targeted modulation was further validated by the loss of efficacy in the ADRB2-knockdown group, underscoring the precision of our Apt-EVs@GP platform.

Beyond the delivery platform, a critical contribution of this work is the comprehensive elucidation of the GATA1/ADRB2 signaling axis in depressive pathology. We identified that chronic stress induces the pathological upregulation of the transcription factor GATA1 in the hippocampus, which in turn represses the expression of the neuroprotective receptor ADRB2. This regulatory relationship was rigorously validated through site-directed mutagenesis of the ADRB2 promoter, showing that mutation of the core GATA1-binding motif (TTTTAATCTTT) completely abolished GATA1-mediated transcriptional repression, thereby precisely defining the molecular site of action. By delivering GP *via* Apt-modified EVs, we restored ADRB2 expression and alleviated depressive behaviors in mice by activating the cAMP/PKA/CREB signaling pathway. Crucially, our study provides robust evidence for the necessity of this signaling axis. The functional necessity of this axis was further confirmed by genetic intervention: shRNA-mediated knockdown of ADRB2 during Apt-EVs@GP treatment markedly abolished therapeutic benefits, including behavioral recovery, normalization of cortisol levels, and neuroprotection. This reversal effect provides compelling evidence that the antidepressant efficacy of Apt-EVs@GP is strictly dependent on functional restoration of ADRB2 signaling rather than nonspecific or off-target mechanisms.

Therapeutically, Apt-EVs@GP exerted broad neuroprotective effects, effectively reversing multiple core pathological features of depression. Behavioral assessments confirmed its efficacy in alleviating despair- and anhedonia-like phenotypes. At the cellular level, Apt-EVs@GP attenuated neuroinflammatory responses, suppressed neuronal apoptosis, and restored synaptic integrity. Although neuronal apoptosis was used in this study as a hallmark of severe CUMS-induced pathology, the GATA1/ADRB2 axis is also a key regulator of synaptic plasticity and CREB-dependent transcription. Therefore, we hypothesize that Apt-EVs@GP may also be effective in milder stress paradigms primarily characterized by synaptic dysfunction rather than overt neuronal loss, a hypothesis that warrants further experimental validation. With respect to safety considerations, ADRB2 is widely expressed in peripheral tissues, including cardiomyocytes, raising potential concerns regarding systemic receptor upregulation. Notably, our biodistribution analyses demonstrated significantly lower accumulation of Apt-EVs@GP in the heart compared with the brain. Consistently, histopathological examination of major organs revealed no detectable toxicity. These findings suggest that the targeted delivery strategy of Apt-EVs@GP effectively minimizes peripheral off-target exposure and associated risks.

In this study, we also evaluated the targeting ability of Apt-EVs@GP using in vivo imaging. The results demonstrated that Apt-EVs@GP accurately targeted and accumulated in the hippocampal region of the mice. This observation is consistent with previous reports on extracellular vesicle–based drug delivery systems, while the key innovation of our approach lies in the incorporation of nucleic acid aptamer modification to further enhance targeting precision and drug release efficiency. Compared with conventional drug delivery platforms, Apt-EVs@GP exhibits clear advantages in terms of targeting specificity and biocompatibility. Moreover, cytotoxicity assays confirmed that Apt-EVs@GP induced minimal cellular toxicity, further supporting its favorable safety profile. Collectively, these properties highlight the potential of EV-based delivery systems as an ideal therapeutic strategy for neurological disorders, particularly those requiring long-term treatment and precise cellular targeting.

Transcriptomic analysis was a central component of this study, enabling the systematic identification of DEGs associated with the CUMS model and revealing the critical involvement of the GATA1/ADRB2 signaling axis in depressive pathology. The transcriptomic data provided a comprehensive molecular framework that facilitated deeper insight into the mechanisms by which Apt-EVs@GP alleviates depressive symptoms. Integration of transcriptomic findings with targeted validation assays, including qRT-PCR and WB analysis, confirmed that Apt-EVs@GP suppresses GATA1 expression, thereby relieving its transcriptional repression of ADRB2. As a classical G protein-coupled receptor (GPCR), ADRB2 regulates CREB (cAMP response element-binding protein) expression through activation of the cAMP/PKA signaling pathway, ultimately triggering the cAMP/PKA/CREB cascade [28]. These findings not only underscore the importance of the GATA1/ADRB2 axis in depression but also offer new molecular targets for the precision treatment of depression.

Nevertheless, several important limitations should be acknowledged to provide a balanced interpretation of our findings. First, GATA1 is classically recognized as a master regulator of hematopoiesis, and its physiological role in the central nervous system remains incompletely defined. Although our transcriptomic and molecular analyses clearly demonstrate pathological upregulation of GATA1 in the hippocampus under chronic stress, the upstream mechanisms driving this induction, such as epigenetic regulation or stress hormone–responsive signaling, remain unresolved. While we identify GATA1 as a stress-responsive transcriptional repressor of ADRB2, future studies are required to systematically map its broader cistrome and regulatory network in neuronal and glial populations. Second, although we delineate a GATA1/ADRB2/cAMP/PKA/CREB signaling axis, depressive pathology is governed by a highly interconnected signaling landscape. It remains possible that cAMP/PKA signaling engages in functional crosstalk with other neurotrophic pathways, such as BDNF/TrkB or PI3K/Akt, which were not comprehensively investigated in the present study. Exploring these interactions may further refine our understanding of downstream signaling integration in depression. Third, while our study utilized oe-GATA1 and sh-ADRB2 to confirm that the GATA1/ADRB2 axis is necessary for the antidepressant action of Apt-EVs@GP, we did not evaluate whether direct genetic modulation alone could fully mimic the therapeutic phenotype in vivo. Our in vitro data (Fig. 7I) showed that GATA1 silencing *via* sh-GATA1 is sufficient to restore ADRB2 levels, and previous studies have suggested that ADRB2 agonists possess antidepressant properties [29]. Therefore, future studies should employ gene-editing tools to directly manipulate this axis in depression models to further validate its sufficiency as a therapeutic target independently of drug delivery. Moreover, the immunogenicity of the nucleic acid Apts, the large-scale production of EVs, and their stability in complex human environments need to be optimized. Future research should integrate single-cell technologies, spatial transcriptomics, and clinical sample analysis to explore additional neuro-immune regulatory axes, providing further evidence for personalized depression treatment while advancing the clinical translation of EV-based drug delivery platforms.

## Conclusion

In conclusion, this study demonstrates that Apt-EVs@GP serves as a potent, targeted nanotherapeutic for depression by reprogramming the GATA1/ADRB2/cAMP axis (Graphic abstract). By restoring ADRB2 expression, this platform effectively reverses neuroinflammation, apoptosis, and synaptic deficits. Despite the acknowledged limitations regarding the non-canonical role of GATA1 and the need for functional electrophysiological validation, our findings provide a novel theoretical framework and a promising precision medicine strategy for treating depression-related hippocampal pathology.

## Materials and Methods

### Instruments and reagents

#### Reagents

All DNA oligonucleotides were synthesized by Sangon Biotech (Shanghai, China) and purified by high-performance liquid chromatography (HPLC). Lyophilized oligonucleotides were dissolved in phosphate-buffered saline (PBS) prior to use. The aptamer sequences were as follows: BI1 scramble, 5′-GTGGCTCGGACGGGTTCGCATGGGAGGCAGACGGGTACAT-3′; BI2 scramble, 5′-GCAAGATTGGAGGAATTTATCAGGGACTCTGGCGTCTATA-3′ (Sangon Biotech, Shanghai, China). Additional reagents included Trypsin (Meryer, M88968-100G), DNase I (Aladdin, D106200-500mg), fetal bovine serum (FBS, Meryer, M85271-100mL), glucose (Meryer, 99%, M93298-1G), poly-L-lysine (Beyotime, ST508), colloidal gold staining reagent (uranyl acetate, Yaji Bio, 99%), Triton-X 100 (Aladdin, BioReagent, T434386-500mL), 5% skim milk powder (Coolaber, Beijing, SL1330-100mL), 0.1% trifluoroacetic acid aqueous solution and acetonitrile (Meryer, M33954-5L and M25342-10L), 0.22 μm sterile filter membranes (Millipore, Aladdin, M2637-B0.22μm-100EA), electroporation buffer (Yuanye, R20981-100mL), Calcein-AM (Macklin, ≥90% (HPLC), C794029-1mg), propidium iodide (PI, Aladdin, ≥98.0%, P266304-25mg), Annexin V-FITC/PI staining kit (BestBio, BB-4101), Bicinchoninic Acid (BCA) protein quantification kit (Aladdin, B665595-1kit), goat anti-mouse IgG (H&L) antibody (Sigma-Aldrich, Cat# SAB4600042, RRID:AB_2532075) for localization of IgG (H&L), polyacrylamide gel (Yuanye), Laemmli buffer (Macklin), mouse monoclonal anti-β-actin (Multi Sciences, Cat#70-ab008-100, RRID:AB_2750915) for localization of β-actin, anti-CD81 (SIGMA, SAB3500454-100UG), rabbit polyclonal anti-TSG101 (Beyotime, Cat# AF8262, RRID:AB_2923333) for localization of TSG101, goat anti-rabbit IgG-HRP (Biosharp, Cat# BL003A, RRID:AB_2827666) for secondary antibody.

#### Instruments

Optima L-100 XP ultracentrifuge (Beckman Coulter, Minggujia Electronic Technology Co., Ltd., Shanghai), qNano nanoparticle analyzer (Izon, Meryer Bio), transmission electron microscopy (TEM, FEI Tecnai F20 TEM D545, FEI Company, USA), enzyme-linked immunosorbent assay (ELISA) microplate reader (ELx800, Bio-Tek, USA, Thermo Fisher), flow cytometer (FACSCalibur, BD Biosciences, USA, Beijing Zhonglilan Biological Technology Co., Ltd.), real-time quantitative PCR system (ABI 7900, Applied Biosystems, USA, Thermo Fisher), Zetasizer Nano ZS (Malvern Instruments, Shanghai Beimi Bio), Tecnai 12 electron microscope (FEI, Hillsboro, Philips), fluorescence microscope (Nikon Eclipse E400, Germany), cold-field emission 12-pole spherical aberration-corrected TEM (JEM-ARM300F2, Shanghai Feliya Industrial Co., Ltd.), Gene Pulser Xcell instrument (Bio-Rad, Jiangsu Kenelfi Experimental Instrument Trading Co., Ltd.).

### Astrocyte culture and stimulation

Primary astrocytes were cultured to generate astrocyte-derived EVs and to evaluate their response to EV-mediated stimulation. Primary astrocytes were isolated from postnatal mouse hippocampal and cortical tissues. The dissected tissues were enzymatically digested with trypsin and deoxyribonuclease I (DNase I) at 37 °C for 15 min, followed by mechanical dissociation. The resulting cell suspension was plated in poly-L-lysine-coated T75 culture flasks and maintained in minimal essential medium (MEM) enriched with 20% FBS (Meryer, M85271-100ML) and glucose (5.5 g/L). After 7 days of culture, microglia were removed by shaking to purify the astrocytes. The purified astrocytes were seeded on glass coverslips and exposed to EVs derived from donor microglia at a 2:1 ratio. To minimize nonspecific cellular activation, cells were maintained in low-serum (1% FBS) medium during stimulation. Following treatment, cells were harvested using TRIzol reagent for subsequent qRT-PCR analysis.

### Extraction and Characterization of Astrocyte-Derived EVs

Culture supernatants from astrocytes were sequentially centrifuged (300 × g, 10 min; 2,000 × g, 10 min; 10,000 × g, 30 min; 4 °C) to remove cells and debris, followed by filtration through a 0.22 μm sterile membrane. EVs were isolated by ultracentrifugation (140,000 × g, 70 min) using an Optima L-100 XP ultracentrifuge, resuspended in PBS, and subjected to a second ultracentrifugation step under identical conditions. Protein concentration was determined using a bicinchoninic acid (BCA) assay, vesicle morphology was examined by TEM, and size distribution and zeta potential were analyzed by NTA. The expression of EV-associated surface markers, including CD9, CD63, CD81, Alix, and tumor susceptibility gene 101 protein (TSG101), was assessed by Western blot (WB).

The size distribution and concentration of EVs obtained from 10,000 × g and 100,000 × g fractions were further assessed by tunable resistive pulse sensing (TRPS) using a qNano instrument (Izon, Christchurch, New Zealand). Measurements were performed by applying a voltage across an electrolyte-filled nanopore, producing transient ionic current blockades as vesicles traversed the pore, with blockade amplitude proportional to vesicle volume. Prior to analysis, nanopores were conditioned with the Izon EV kit to minimize vesicle aggregation, and EV samples were suspended in measurement buffer. NP300 nanopores (150–600 nm) were used for microvesicle analysis, whereas NP150 nanopores (85–300 nm) were used for EV analysis. Calibration was conducted using standard beads (GPC200 and GPC150), and data acquisition and analysis were performed using Izon Control Suite software (version 3.2).

### Nucleic acid Apt modification of EVs to form Apt-Modified Astrocyte-Derived EVs (Apt-EVs)

The EVs (3 μg, 60 μL) were incubated with cholesterol-conjugated Apts (chol-Apt, 10 μM) at room temperature (RT) for 5 min. The mixture was washed three times with PBS using a 100 kDa molecular weight cutoff (MWCO) filter (Millipore Sigma) at 5000 × g for 5 min to remove unbound oligonucleotides. The hydrodynamic diameter and zeta (ζ) potential of Apt-EVs were measured using a Litesizer 500 (Anton Paar, Austria) at 25 °C.

### Conjugation Efficiency and Apt Density on EVs

Unreacted Apts in the supernatant were quantified by gradient HPLC using an Agilent 1260 system equipped with a Phenomenex C18 column (5 μm, 250 mm × 4.6 mm). Chromatographic separation was performed at 40 °C with ultraviolet (UV) detection at 280 nm and a flow rate of 1 mL/min. The mobile phase consisted of solvent A (0.1% trifluoroacetic acid in water) and solvent B (0.1% trifluoroacetic acid in acetonitrile). The gradient program was set as follows: 5% B for 25 min, a linear increase to 30% B, followed by an increase to 100% B over 8 min, held for 40 min, and then returned to 5% B. The retention time of Apts was 12.8 min, and calibration curves showed good linearity over the range of 2–200 μg/mL (R^2^ = 0.9935).

Conjugation efficiency was calculated by comparing the HPLC peak area of residual Apts in the supernatant with that of standard Apt solutions at equivalent concentrations and was expressed as a percentage of the total input Apts. Apt density on EVs was determined as the ratio of conjugated Apt molecules to EV particle number. To quantify Apt loading, 3’-FAM-labeled Apts were conjugated to EVs by vortex mixing (500 rpm, 5 min). The product was divided into two portions: one for EV concentration measurement using the NanoSight NS300 (Malvern) and the other for Apt quantification following treatment with 5% Triton X-100 for 5 min. Fluorescence intensity was recorded with a microplate reader (BioTek Synergy H4, excitation 494 nm, emission 520 nm) and compared with a standard curve of known FAM-Apt concentrations. The average number of Apts per EV was calculated by dividing the molar concentration of Apts by EV count.

### Electroporation-mediated Loading of GP

EVs and GP were mixed at a 1:1 weight ratio and diluted in electroporation buffer to a final concentration of 0.5 mg/mL. The mixture was transferred into 0.4 mm electroporation cuvettes and electroporated using a Gene Pulser Xcell system (Bio-Rad, Hercules, CA, USA) under the following conditions: 400 V, 125 μF, and a pulse duration of 10–15 ms. Unencapsulated GP was removed by washing twice with ice-cold PBS, followed by ultracentrifugation (140,000 × g, 70 min, 4 °C). The resulting pellet was finally resuspended in PBS for subsequent experiments.

### Characterization of EVs, Apt-EVs, and Apt-EVs@GP

Dynamic light scattering (DLS) was performed on a Zetasizer Nano ZS (Malvern Instruments) at a scattering angle of 173° and a laser wavelength of 633 nm at 25 °C. The polydispersity index (PDI) and zeta potential were measured, and each sample (EVs, Apt-EVs, and Apt-EVs@GP) was analyzed in triplicate.

### NTA

NTA was performed at RT using the NanoSight NS300 system (Malvern, UK). Samples were diluted in PBS to 10^7^–10^9^ particles/mL to ensure accurate detection. Each measurement included three 30 s video captures with a 10 s interval. Data acquisition and analysis were conducted in real time with NTA software (version 3.0), and particle size distribution and concentration were calculated from Brownian motion profiles.

### Cryo-TEM

The morphology of EVs, Apt-EV, and Apt-EVs@GP was examined using Cryo-TEM. Briefly, 10 μL of each sample (2 × 10^11^ particles/mL) was applied onto a copper grid, blotted to generate a thin liquid film, and rapidly plunge-frozen in liquid ethane, followed by cooling in liquid nitrogen to 90 K. Imaging was performed using a Tecnai 12 transmission electron microscope (FEI, USA) operated at an accelerating voltage of 120 kV.

### Physical stability

To assess the stability, EVs and Apt-EVs were stored in PBS at 4 °C for up to 10 weeks. Size, PDI, and zeta (ζ) potential were monitored over time using.

### Serum stability assessment

To evaluate the serum stability of Apt-EVs@GP, free Apt, Apt-EVs, or Apt-EVs@GP were incubated with 50% FBS. At various time points, aliquots from each group were subjected to agarose gel electrophoresis, and the results were visualized under UV light.

### WB analysis of EV-specific surface markers

After treatment, culture dishes were placed on ice and washed twice with PBS. Proteins were extracted using radioimmunoprecipitation assay (RIPA) buffer (50 μL) on ice for 30 min, followed by centrifugation (12,000 × g, 10 min) to remove insoluble debris. Protein concentration was measured using a BCA assay. Equal protein amounts (60 μg) were separated by SDS-PAGE and transferred onto PVDF membranes. Membranes were blocked in 5% non-fat milk in Tris-buffered saline with Tween 20 (TBST) for 1 h, incubated overnight at 4 °C with primary antibodies, washed three times, and then incubated with IRDye 800 W-conjugated goat anti-mouse IgG (H&L) antibody (0.33 μg/mL) for 2 h. After washing, signals were detected using the Odyssey infrared imaging system (LI-COR, NE).

### WB Analysis of Astrocyte-Derived EV Markers

Total protein concentrations from cells, astrocyte-derived EVs, and Apt-EVs were quantified in triplicate using the Pierce™ BCA Protein Assay Kit. Soluble proteins extracted with 1× RIPA buffer were mixed with 5× Laemmli buffer under reducing or non-reducing conditions. Equal amounts of protein (25 μg per lane) were separated on 12% polyacrylamide gels by electrophoresis (120 V, 80 min) and transferred onto PVDF membranes (200 mA, 2 h). Membranes were blocked in 5% non-fat milk in PBS containing 0.05% Tween-20 (PBS-T) for 1 h, washed three times with PBS-T, and incubated with primary antibodies (mouse monoclonal anti-β-actin, anti-CD81, and rabbit polyclonal anti-TSG101) at RT for 1 h. After washing, membranes were incubated with horseradish peroxidase (HRP)-conjugated goat anti-rabbit IgG (H&L) for 1 h, washed, and developed with Amersham™ ECL™ Prime reagent. Protein bands were visualized using the ImageQuant LAS3000 system (Bio-Rad), and images were processed with Image Lab software (version 6.0.1).

### Assessment of GP loading

Drug loading was assessed at different incubation times (0.5, 1, 1.5, 2, 3, and 4 h). Free GP was quantified by HPLC. Briefly, EV–GP mixtures were ultracentrifuged (100,000 × g, 1 h, 4 °C) to remove free drug, and the resulting EV pellets were washed with PBS and subjected to a second ultracentrifugation under identical conditions. Supernatants containing free GP were analyzed using an Agilent Luna C18 column (150 mm × 4.6 mm). The mobile phase consisted of MilliQ water adjusted to pH 2.6 with 85% phosphoric acid and acetonitrile (74:26, v/v) at 1.0 mL/min, with the column maintained at 40 °C. Fluorescence detection was performed at λ_ex_ = 475 nm and λ_em_ = 555 nm. Encapsulation efficiency (EE) and loading capacity (LC) were calculated as: EE (%) = (encapsulated GP / total GP added) × 100; LC (%) = (encapsulated GP/total EV weight) × 100.

### In vitro drug release characteristics

Drug release was studied under physiological (PBS, pH 7.4) and acidic conditions (acetate buffer, pH 5.0). EV@GP or Apt-EVs@GP (1 mL, 200 μg/mL) were dialyzed in 3.5 kDa membranes at 37 °C with stirring (150 rpm). At predetermined time points, 500 μL of release medium was withdrawn and replaced with fresh buffer. Samples were analyzed by HPLC, and cumulative release (%) was calculated as: Drug release (%) = (GP in release medium / total GP in vesicles) × 100.

### Evaluation of cellular targeting of Apt-EVs

Cellular uptake of Apt-EVs was evaluated in astrocytes, with unmodified EVs as controls. Astrocytes (2 × 10⁵ cells/mL) were seeded onto glass coverslips and incubated for 24 h. PKH67-labeled EVs or Apt-EVs (4 × 10⁴ particles/cell) were added and incubated for 0.5, 1, or 2 h. Following incubation, cells were stained with Hoechst 33342 (20 min, 37 °C), washed with PBS, and fixed with 4% paraformaldehyde (PFA) for 15 min. Coverslips were mounted with Citifluor solution, and uptake was visualized using a Nikon Eclipse E400 fluorescence microscope. Fluorescence intensity was quantified with Fiji software. For competition assays, astrocytes were pre-incubated with 0.5 mM free Apt for 15 min before the addition of Apt-EVs, and uptake was subsequently analyzed.

### Evaluation of Neuronal Targeting of Apt-EVs

To assess neuronal targeting, only Cy5-labeled Apt-EVs (Apt-EVs-Cy5) were tested. Cy5, Apt-EVs-Cy5, and unmodified EVs-Cy5 were intraperitoneally injected into female C57BL/6 J mice. After 24 h, mice were anesthetized and perfused with saline followed by 4% PFA in PBS. Brains were dissected, post-fixed overnight in 4% PFA, and cryoprotected in 30% sucrose. Coronal brain sections (40 μm) were permeabilized with 0.3% Triton X-100 in PBS for 1 h, treated with 3% H_2_O_2_ for 10 min, and blocked with 10% goat serum. Sections were then incubated with an anti-MAP2 primary antibody (1:400; Abcam, UK) for 2 h at RT. After PBS washes, sections were incubated with IRDy594- or IRDy488-conjugated secondary antibodies (1:500; LI-COR, USA) for 1 h. Fluorescence was visualized with a Leica confocal microscope (Leica Microsystems, Japan).

### In vitro cytotoxicity assay

The cytotoxicity of Apt-EVs@GP toward astrocytes was assessed using the Cell Counting Kit-8 (CCK-8) assay. Astrocytes (1 × 10^4^ cells per well) were seeded into 96-well plates and cultured for 24 h. Cells were subsequently treated with Apt-EVs@GP at concentrations ranging from 0 to 50 μg/mL for 4 h. Cell viability was determined by measuring absorbance at 450 nm using a microplate reader (ELx800, Bio-Tek, USA).

### Cell Treatment and Apoptosis Detection

For live/dead staining, astrocytes (1 × 10⁵ cells/well) were seeded in 6-well plates and cultured for 24 h. Cells were exposed to PBS, Apt-EVs, GP, or Apt-EVs@GP (25 μg/mL) with or without 808 nm laser irradiation (0.8 W/cm^2^, 5 min). After 12 h, cells were washed with PBS and subjected to live/dead staining using Calcein-AM (2.0 μM) and propidium iodide (PI; 4.5 μM) for 30 min, followed by confocal microscopy imaging. For apoptosis analysis, cells receiving identical treatments were harvested by trypsinization, washed, and stained with Annexin V-FITC/PI. The proportion of apoptotic cells was subsequently quantified by flow cytometry (FCM).

### NeuN immunofluorescence/TUNEL double staining

Mice were randomly assigned to the Saline, Apt-EVs@GP, Apt-EVs, GP, or EV groups and treated via tail vein injection once daily for 21 days (GP dose: 50 mg/kg). After treatment, brains were harvested on ice, fixed in 4% PFA (4–6 h, 4 °C), cryoprotected in sucrose, embedded in optimal cutting temperature (OCT) compound, and sectioned at a thickness of 40 μm. Brain sections were washed with TBST, subjected to antigen retrieval, and blocked with 10% donkey serum (37 °C, 30 min). Sections were then incubated with an anti-NeuN monoclonal antibody for 2 h at RT, followed by incubation with the appropriate secondary antibody (37 °C, 1 h) in the dark. TUNEL staining was then performed, followed by DAPI counterstaining. Fluorescence signals were observed under a fluorescence microscope, and the percentage of TUNEL-positive cells was quantified using ImageJ software.

### CUMS model

Male C57BL/6 mice (18–22 g, strain code: 219) were purchased from Beijing Vital River Laboratory Animal Technology Co., Ltd. Animals were housed under standardized conditions (12 h light/dark cycle, 23 ± 1 °C, 45 ± 5% relative humidity) with ad libitum access to food and water. The CUMS model was established over a 42-day period by randomly applying one or two stressors per day to prevent stress habituation. Stressors included cold-water swimming (4 °C, 5 min), food and water deprivation (24 h), reversal of the light/dark cycle (24 h), wet bedding (300 mL water for 8–20 h), restraint (6 h), cage tilting (45°, 12 h), and cage rotation (30 min to 2 h) [30].

### Animal grouping and intervention

Mice were randomly assigned to the following groups: control, CUMS, Apt-EVs@GP, Apt-EVs, EVs, Fluoxetine (positive control) [31], Apt-EVs@GP + oe-GATA1, and Apt-EVs@GP + sh-ADRB2. Apt-EVs@GP (GP equivalent: 50 mg/kg) was administered *via* tail vein injection once daily for 21 days. During treatment, CUMS mice continued to receive daily stress stimulation. For genetic modulation, lentiviral vectors encoding oe-GATA1 or sh-ADRB2 (1 × 10⁹ TU/mL, GeneChem, China) were delivered into the hippocampal region by intracerebroventricular injection. The dentate gyrus (DG) and cornu ammonis (CA) subregions, located near the inferior horn of the lateral ventricle, facilitated exchange between cerebrospinal fluid and hippocampal interstitial space [32]. All animal experiments were approved by the Animal Ethics Committee of Yongchuan Hospital of Chongqing Medical University and conducted in accordance with the *Guide for the Care and Use of Laboratory Animals*.

### In vivo imaging system

To assess biodistribution, Cy5-labeled Apt-EVs@GP and free GP were administered *via* tail vein injection. After 24 h, mice were anesthetized with 2% isoflurane (Antai Biotechnology, China) and imaged using the In Vivo Imaging System Spectrum (IVIS, PerkinElmer, USA). Fluorescence intensity was quantified with Living Image 4.5 software (PerkinElmer, USA).

### Fluorescence Imaging

To confirm brain targeting, mice were sacrificed 24 h after Cy5-labeled Apt-EVs@GP injection. Brains were harvested and fixed in 10% neutral buffered formalin (Beyotime, China) for 48 h, cryoprotected in 30% sucrose, and cryosectioned at a thickness of 10 μm using a Leica CM1950 cryostat (Leica Microsystems, Germany). Tissue sections were washed with PBS and counterstained with DAPI (1:1000; Solarbio, China) for 10 min. Fluorescence images were acquired using a Leica DMi8 fluorescence microscope (Leica Microsystems, Germany) to assess the distribution of Apt-EVs@GP in the hippocampal region.

### Serum biochemical marker analysis

To evaluate the biocompatibility of Apt-EVs@GP, tail vein blood was collected, and serum was separated. ALT, AST, BUN, and CREA levels were measured using an automatic biochemical analyzer (Mindray BS-240, China). The ALT and AST kits (Catalog No. C009-2-1, C010-2-1), BUN kit (Catalog No. C013-2-1), and CREA kit (Catalog No. C011-2-1) were purchased from Nanjing Jianchen Bioengineering Institute (China).

### Hematoxylin and Eosin (H&E) staining

Mouse hippocampal tissue as well as heart, liver, spleen, lung, and kidney samples were fixed in 10% neutral buffered formalin for 48 h, paraffin-embedded, and sectioned at a thickness of 5 μm using a microtome (Leica Microsystems, Germany). The tissue sections were stained with H&E (Solarbio, China) and observed for histopathological changes under an optical microscope (Olympus BX53, Olympus, Japan). Tissue structural analysis was conducted using CaseViewer software (3DHISTECH, Hungary).

### Transcriptome sequencing

Total RNA was extracted from hippocampal tissues of the Normal, CUMS, and Apt-EVs@GP treatment groups using TRIzol reagent (Invitrogen, USA). Approximately 50 mg of tissue was homogenized, followed by chloroform extraction, isopropanol precipitation, and ethanol washing. RNA pellets were resuspended in RNase-free water, and RNA purity was evaluated using a NanoDrop 2000 spectrophotometer (Thermo Fisher Scientific, USA). Samples with OD260/280 ratios between 1.8 and 2.0 were selected for library construction. Ribosomal RNA was removed using the Ribo-Zero Kit (Illumina, USA), and sequencing libraries were prepared with the NEBNext Ultra RNA Library Prep Kit (New England Biolabs, USA). Libraries were sequenced on an Illumina NovaSeq 6000 platform (Illumina, USA) to generate approximately 6 Gb of 150 bp paired-end reads per sample. Raw sequencing data were subjected to quality control using FastQC (v0.11.9), and only high-quality reads were retained for subsequent analysis.

### Principal Component Analysis (PCA)

Normalized gene expression matrices were generated using DESeq2 (v1.38.0). PCA was performed to evaluate overall gene expression patterns among Normal, CUMS, and Apt-EVs@GP groups. PCA scatter plots were visualized using the ggplot2 package (v3.3.6).

### Differential expression analysis

Clean reads were aligned to the mouse reference genome (GRCm39, Ensembl) using HISAT2 (v2.2.1). Gene expression was quantified with StringTie (v2.1.4) and expressed as fragments per kilobase of transcript per million mapped reads (FPKM). Differentially expressed genes (DEGs) were identified with DESeq2 using |log_2_FoldChange | ≥ 1 and adjusted *p*-value < 0.05. DEGs were compared between CUMS vs. Normal and Apt-EVs@GP vs. CUMS groups, and the intersecting genes were selected for further analysis. Results were visualized using volcano plots and heatmaps. Volcano plots illustrated log_2_FoldChange versus −log_10_ adjusted *p*-values, with upregulated genes shown in red, downregulated genes in blue, and non-significant genes in gray. Heatmaps depicted the expression patterns of significant DEGs across groups based on standardized FPKM values, with hierarchical clustering applied to reveal intergroup relationships.

### Chromatin Immunoprecipitation (ChIP) assay

ChIP-qPCR was performed to verify direct binding of GATA1 to the ADRB2 promoter. Human embryonic kidney 293 T cells (ATCC, USA) were transfected with either control plasmids or GATA1 overexpression plasmids (GenePharma, China), forming oe-NC and oe-GATA1 groups. After 48 h, cells were fixed with 0.75% formaldehyde (Sigma-Aldrich, USA) for 10 min, and the reaction was quenched with 125 mM glycine. Chromatin was isolated with ChIP lysis buffer (Cell Signaling Technology, USA) and fragmented by sonication to 200–500 bp. Chromatin was incubated with a rabbit monoclonal anti-GATA1 antibody (Abcam, Cat# ab181544, RRID:AB_2920794) and pulled down using Protein A/G magnetic beads (Thermo Fisher Scientific, USA). IgG served as a negative control. Immunoprecipitated DNA was purified (Qiagen, Germany), and enrichment of the ADRB2 promoter region was quantified by qPCR. Input DNA was used for normalization.

### Dual-Luciferase reporter assay

To evaluate GATA1 regulation of ADRB2 transcription, reporter plasmids containing either the wild-type (WT, sequence: TTTTAATCTTT) or mutated (MUT, sequence: TTTTAACGTTT) ADRB2 promoter were constructed (GenePharma, China). A GATA1 overexpression plasmid (oe-GATA1, GenePharma, China) was used. Human 293 T cells were transfected with 0.5 µg of WT or MUT plasmid, 0.05 µg of Renilla luciferase control plasmid, and 1 µg of oe-GATA1 or control plasmid using Lipofectamine 3000 (Invitrogen, USA). After 48 h, firefly and Renilla luciferase activities were measured using the Dual-Luciferase Reporter Assay System (Promega, USA). Relative luciferase activity was calculated as the firefly-to-Renilla ratio, and promoter activities were compared among groups.

### Immunofluorescence (IF) staining in 293 T cells

Co-localization of GATA1 and ADRB2 was assessed by IF staining. Human embryonic kidney 293 T cells (CLS Cat#305117, RRID:CVCL_1926) were fixed with 4% formaldehyde (Sigma-Aldrich, USA) for 10 min and permeabilized with 0.2% Triton X-100 (Beyotime, China). Cells were incubated overnight at 4 °C with primary antibodies: anti-GATA1 (1:200, Invitrogen, Cat# MA5-15638) and anti-ADRB2 (1:100, Invitrogen, Cat# PA5-86339). After PBS washes, cells were incubated for 1 h with secondary antibodies: goat anti-mouse IgG H&L (Alexa Fluor 488, 1:100, Abcam Cat# ab150113, RRID:AB_2576208) and goat anti-rabbit IgG H&L (Alexa Fluor 594, 1:100, Abcam Cat# ab150080, RRID:AB_2650602). Nuclei were counterstained with DAPI. Images were acquired using a laser scanning confocal microscope (Leica Microsystems, Germany).

### Open Field Test (OFT)

Locomotor and exploratory behaviors were assessed using the OFT. Mice were individually placed in the center of a square arena (50 × 50 × 40 cm) and allowed to explore for 5 min. Total distance traveled and mean movement speed were automatically quantified using a video-tracking system.

### Forced Swim Test (FST)

Depressive-like behavior was evaluated using the FST. Mice were individually placed in transparent cylinders (10 cm diameter, 50 cm height) containing water maintained at 25 °C to a depth of 40 cm, preventing escape or tail support. Immobility time during a 5 min test period was automatically recorded using a video-tracking system.

### Tail Suspension Test (TST)

The TST was conducted to assess depressive-like behavior. Mice were suspended by the tail using adhesive tape at a height of 50 cm above the floor, and immobility time was recorded over a 5 min test period.

### Sucrose Preference Test (SPT)

Anhedonia was evaluated with the SPT. During the habituation phase, mice were provided with two bottles containing 1% sucrose solution for 24 h, followed by 24 h of access to one bottle of 1% sucrose and one bottle of water, with bottle positions alternated to avoid side bias. For the test phase, mice were deprived of food and water for 12 h and then given access to one bottle of 1% sucrose solution and one bottle of water for 12 h, with bottle positions switched halfway through the test. Fluid consumption was measured, and sucrose preference was calculated as follows: Sucrose preference (%) = [Sucrose intake / (Sucrose intake + Water intake)] × 100%.

### Three-Chamber Social Interaction Test (ST)

The ST was performed to assess sociability and preference for social novelty. The apparatus consisted of a white rectangular box (100 × 50 × 40 cm) divided into three interconnected chambers, with identical wire cages (10 cm diameter) placed in the left and right chambers. Mice were acclimated to the testing room for 1 h before the experiment and allowed to habituate in the center chamber for 10 min. In Phase I (sociability), a novel mouse was placed in one wire cage while the other cage remained empty, and the test mouse was allowed to explore freely for 10 min. In Phase II (social novelty), the familiar mouse was placed back into one cage, and a new unfamiliar mouse was introduced into the other cage, followed by an additional 10 min exploration period. The time spent in each chamber and the number of chamber entries were recorded. The apparatus was cleaned with 75% ethanol between trials. Behavioral data, including locomotor activity, chamber entries, and cumulative exploration time, were analyzed using Tracking Master V3.0 software.

### qRT-PCR analysis

Total RNA was isolated from mouse brain tissue using TRIzol reagent (Invitrogen, USA). Complementary DNA (cDNA) was synthesized with a reverse transcription kit (Takara Bio, Japan), and quantitative real-time PCR was performed using SYBR Green PCR Master Mix (Thermo Fisher Scientific, USA) on a QuantStudio 5 system (Applied Biosystems, USA). Gene expression levels were normalized to GAPDH, and primer sequences are provided in Table S1. Relative expression levels were calculated utilizing the 2^-ΔΔCt^ method.

### WB analysis

Total protein was extracted from mouse brain tissue using RIPA lysis buffer (Beyotime, China) supplemented with protease inhibitors (Roche, Switzerland). Protein concentration was determined using the BCA assay (Thermo Fisher Scientific, USA). After separation on 10% SDS-PAGE, proteins were transferred to a PVDF membrane (Millipore, USA). The membrane was incubated overnight with primary antibodies: anti-GATA1 (1:1000, ab300613, Abcam, UK), anti-ADRB2 (1:1000, Abcam Cat# ab182136, RRID:AB_2747383), anti-PKA (1:1000, Cell Signaling Technology Cat# 5842, RRID:AB_10706172), anti-CREB (1:1000, Cell Signaling Technology Cat# 9197, RRID:AB_331277), anti-TNF-α (1:1000, Abcam Cat# ab1793, RRID:AB_302615), anti-IL-6 (1:1000, Cell Signaling Technology Cat# 12912, RRID:AB_2798059), and anti-β-tubulin (1:1000, Abcam Cat# ab6046, RRID:AB_2210370). After washing, membranes were incubated with an HRP-conjugated secondary antibody (1:5000; Abcam, Cat# ab205718, RRID: AB_2819160) for 1 h. Protein bands were visualized using an ECL detection kit (Bio-Rad, USA) and analyzed with the ChemiDoc system (Bio-Rad, USA). Full and uncropped western blots are provided in the Supplemental Material.

### Serum cortisol detection

Serum cortisol levels were quantified using a commercial ELISA kit (EIACORT; Thermo Fisher Scientific, USA). Blood samples were collected via tail vein puncture and centrifuged (3,000 rpm, 10 min, 4 °C) to obtain serum. Cortisol concentrations were determined according to the manufacturer’s instructions. The assay detection range was 0.5–50 ng/mL, and absorbance was recorded at 450 nm using a microplate reader (BioTek, USA).

### Nissl staining

Hippocampal tissue sections were deparaffinized in xylene, rehydrated through a graded ethanol series (100%, 95%, 85%, 70%), and subjected to antigen retrieval in 0.01 M sodium citrate buffer (pH 6.0). After cooling, sections were stained with Nissl solution (G1432, Solarbio, China) for 10 min and rinsed with distilled water. Sections were dehydrated, cleared in xylene, and mounted. Nissl body distribution was observed using a Zeiss optical microscope (Zeiss, Germany), and ImageJ software was used to quantify Nissl body number and neuronal density.

### IF Staining in Brain Tissue

Brain sections were fixed with 4% PFA (Sigma-Aldrich, USA) for 10 min and permeabilized with 0.1% Triton X-100 in PBS for 15 min. Blocking was performed with 1% BSA in PBS for 1 h. Sections were incubated overnight at 4 °C with primary antibodies against Iba-1 (1:100, Abcam Cat# ab178847, RRID:AB_2832244) and GFAP (1:100, CST Cat# 12389, RRID:AB_2631098). After washing, Alexa Fluor® 594-conjugated goat anti-rabbit IgG (1:100, Thermo Fisher Cat# A-11012, RRID:AB_2534079) and Alexa Fluor® 488-conjugated goat anti-rabbit IgG (1:100, Thermo Fisher Cat# A-11008, RRID:AB_143165) were applied for 1 h in the dark. Nuclei were counterstained with DAPI (1:1000, CST, USA) for 10 min. Images were captured using a Zeiss LSM 980 confocal microscope, and ImageJ was used to quantify Iba-1- and GFAP-positive cells.

### TUNEL staining

Paraffin-embedded brain sections were deparaffinized in xylene and rehydrated through a graded ethanol series (100%, 95%, 85%, and 70%). After washing with PBS, antigen retrieval was performed by incubation with Proteinase K at 37 °C for 20 min. Sections were then incubated with TUNEL reaction mixture (Roche, Cat# 11684795910, Switzerland) at 37 °C for 1 h in the dark. Following PBS washes, nuclei were counterstained with DAPI for 10 min, and sections were mounted. Fluorescence images were acquired using a Zeiss fluorescence microscope (Germany), and the percentage of TUNEL-positive cells was quantified using ImageJ software.

### TEM of Synaptic Structure

Hippocampal brain tissue (∼1 mm^3^) was fixed in 2.5% glutaraldehyde (Electron Microscopy Sciences, USA) at 4 °C for 24 h, post-fixed with 1% osmium tetroxide (Sigma-Aldrich, USA) for 2 h, and dehydrated through graded ethanol and acetone. Samples were embedded in epoxy resin (Epon 812, Electron Microscopy Sciences, USA), sectioned at 70 nm with an ultramicrotome (Leica EM UC7, Leica Microsystems, Germany), and stained with 2% uranyl acetate (20 min) and 2.6% lead citrate (15 min). Synaptic ultrastructure was examined under TEM.

### Statistical analysis

Data are expressed as mean ± standard error of the mean (SEM) from ≥ 3 independent replicates. Statistical analyses were performed with GraphPad Prism 9.0 (GraphPad Software, USA). Normality was assessed prior to testing. Two-tailed unpaired t-tests were applied for two-group comparisons, while one-way analysis of variance (ANOVA) with Tukey’s post hoc test was used for multiple-group comparisons. Repeated-measures ANOVA was used for time- or dose-dependent variables. Chi-square tests were applied to categorical data. A *p*-value < 0.05 was considered statistically significant. Significance levels are indicated as *p < 0.05, **p < 0.01, ***p < 0.001, and ****p < 0.0001.

### Nomenclature of targets and ligands

Nomenclature conforms to the IUPHAR/BPS Guide to Pharmacology. ADRB2 (β2-adrenergic receptor): indexed in the Guide to Pharmacology (link added in the revised manuscript). GATA1 (gene, transcription factor): not a classical pharmacological target and therefore not indexed in the Guide. Geniposide (natural product): not currently indexed in the Guide.

### Summary

#### What is already known

- Astrocyte-derived extracellular vesicles can deliver neuroprotective molecules.
- GATA1/ADRB2 signaling is implicated in depression pathophysiology.

#### What this study adds

- Aptamer-modified vesicles (Apt-EVs@GP) target hippocampal neurons effectively.
- Apt-EVs@GP relieve depression-like behavior by restoring ADRB2/cAMP signaling.

## Supplementary information

Supplementary figure and table legends

Figure S1. Characterization and optimization of Apt-EVs.

Figure S2. Stability and characterization of Apt-EVs@GP compared to native EVs.

Figure S3. Effects of Apt-EVs@GP on neurons.

Figure S4. Differential gene screening and functional enrichment analysis.

Figure S5. Regulation of differential gene expression by Apt-EVs@GP in CUMS mice.

Figure S6. Schematic of in vivo experimental workflow for mice treated with Apt-EVs@GP

Figure S7. Effects of Apt-EVs@GP on the expression of inflammatory factors via the GATA1/ADRB2 signaling pathway.

Figure S8. Effects of Apt-EVs@GP on neuroinflammation, cell apoptosis, and synaptic damage in brain tissue of CUMS mice via GATA1/ADRB2 regulation.

Figure S9. Effects of Apt-EVs@GP on synaptic damage in CUMS mice via GATA1/ADRB2 regulation.

Table S1. RT-qPCR primer sequences.

The full uncropped Gels and Blots images

## Data Availability

The data that support the findings of this study are available from the corresponding author upon reasonable request. Some data may not be made available because of privacy or ethical restrictions.
