# Supplementary material for: GATA1 repression-mediated ADRB2/cAMP/PKA signaling activation underlies the antidepressant effects of targeted geniposide delivery
Source: Cell Death Discov. 2026 Jun 11;12:338. doi: 10.1038/s41420-026-03190-z (PMC13482994; doi:10.1038/s41420-026-03190-z)
Supplement: Supplementary file 1 [file 41420_2026_3190_MOESM11_ESM.docx]

**Table S1. RT-qPCR primer sequences.**

| **Gene** | **Primer sequences (5’-3’)** |
| --- | --- |
| GATA1 | F: GTCCAAGGGATCTTGGTCCTG |
|  | R: GGCCCCTAGACCAGGAAAATC |
| ADRB2 | F: CTGGTTGGGCTACGTCAACT |
|  | R: TCCGTTCTGCCGTTGCTATT |
| CREB | F: TGTACCACCGGTATCCATGC |
|  | R: TGGATAACGCCATGGACCTG |
| TNF-α | F: GATCGGTCCCCAAAGGGATG |
|  | R: CCACTTGGTGGTTTGTGAGTG |
| IL6 | F: TGATGGATGCTACCAAACTGGA |
|  | R: TGTGACTCCAGCTTATCTCTTGG |
| GAPDH | F: CCCTTAAGAGGGATGCTGCC |
|  | R: TACGGCCAAATCCGTTCACA |

Note: F: Forward; R: Reverse.
