# Supplementary material for: GATA1 repression-mediated ADRB2/cAMP/PKA signaling activation underlies the antidepressant effects of targeted geniposide delivery
Source: Cell Death Discov. 2026 Jun 11;12:338. doi: 10.1038/s41420-026-03190-z (PMC13482994; doi:10.1038/s41420-026-03190-z)
Supplement: Supplementary file 2 [file 41420_2026_3190_MOESM12_ESM.docx]

Figure 7I-1

Figure 7I-2

Figure 7I-3

Figure 8A-1

Figure 8A-2

Figure 8A-3

Figure 8A-4

Figure 8A-5

Figure S1E-1

Figure S1E-2

Figure S1E-3

Figure S1E-4

Figure S1E-5

Figure S1E-6

Figure S6F-1

Figure S6F-2

Figure S6F-3
