# Supplementary material for: GATA1 repression-mediated ADRB2/cAMP/PKA signaling activation underlies the antidepressant effects of targeted geniposide delivery
Source: Cell Death Discov. 2026 Jun 11;12:338. doi: 10.1038/s41420-026-03190-z (PMC13482994; doi:10.1038/s41420-026-03190-z)
Supplement: Supplementary file 3 [file 41420_2026_3190_MOESM1_ESM.docx]

**Supplementary figure and table legends**

**Figure S1. Characterization and optimization of Apt-EVs.**

Note: (A) UV-Vis spectra comparing the absorption peaks of EVs and Apt-EVs@GP; (B) TIRFM image of FAM-Chol-Apt-labeled EVs (scale bar: 25 μm); (C) Optimization of GP loading time (0.5, 1, 1.5, 2, 3, and 4 h); (D) Fluorescence labeling to verify the amount of Apt modification; (E) Western blot analysis of EV marker expression after modification; (F) PKH67-labeled EVs (green) showing uptake efficiency in astrocytes at different time points (scale bar: 25 μm); (G) Quantitative

analysis of data from panel F. **p* < 0.05, ***p* < 0.01, ****p* < 0.001 indicate significant differences between groups.

**Figure S2. Stability and characterization of Apt-EVs@GP compared to native EVs.**

Note: (A) Multimode reader results comparing Apt-modified and unmodified (both FAM-labeled) EVs after co-incubation; (B) Agarose gel electrophoresis showing serum stability of Apt-EVs@GP, EV@GP, and free GP; (C) TEM images of native EVs and Apt-EVs stored in PBS at room temperature for 0 h, 72 h, and 30 days; (D) DLS measurements of native EVs stored in PBS at room temperature for 72 h; (E) DLS measurements of Apt-EVs stored in PBS at room temperature for 72 h.

**Figure S3. Effects of Apt-EVs@GP on neurons.**

Note:(A) NeuN immunofluorescence/TUNEL double staining showing the effect of Apt-EVs@GP on neuronal apoptosis. Red: TUNEL; Green: NeuN; Yellow (merge): apoptotic neurons; (B) Quantification of apoptotic neurons shown in panel A; (C) Neuronal targeting of Apt-EVs. Green: MAP2 (neuronal marker); Blue: nuclei; Red: Cy5-labeled Apt-EVs.

**Figure S4. Differential gene screening and functional enrichment analysis.**

Note: (A-B) Volcano plots showing the distribution of DEGs between the CUMS and normal mice (A) and between the Apt-EVs@GP-treated and CUMS groups (B); (C) Venn diagram showing the overlap of DEGs between the CUMS vs. normal mice and the Apt-EVs@GP vs. CUMS group; (D) KEGG pathway enrichment analysis of overlapping genes; (E) GO functional enrichment analysis annotating the molecular functions of overlapping genes.

**Figure S5. Regulation of differential gene expression by Apt-EVs@GP in CUMS mice.**

Note: (A) PCA showing the distribution of gene expression in the normal, CUMS, and Apt-EVs@GP treatment groups; (B) Venn diagram displaying the overlap between upregulated genes in the CUMS vs. normal mice and downregulated genes in the Apt-EVs@GP vs. CUMS group; (C) Heatmap illustrating the expression patterns of 55 key genes across different groups; (D) Venn diagram showing the overlap between downregulated genes in the CUMS vs. normal mice and upregulated genes in the Apt-EVs@GP vs. CUMS group; (E) Heatmap displaying the expression patterns of 8 key genes across different groups.

**Figure S6. Schematic of in vivo experimental workflow for mice treated with Apt-EVs@GP**

**Figure S7. Effects of Apt-EVs@GP on the expression of inflammatory factors via the GATA1/ADRB2 signaling pathway.**

Note: (A-E) RT-qPCR analysis of mRNA levels for GATA1, ADRB2, CREB, TNF-α, and IL-6 in the brain tissues of each group; (F-H) Western blot analysis of TNF-α and IL-6 protein levels in brain tissues, including quantification. Data are presented as mean ± SEM. **p* < 0.05, ***p* < 0.01, ****p* < 0.001, *****p* < 0.0001. N = 6.

**Figure S8. Effects of Apt-EVs@GP on neuroinflammation, cell apoptosis, and synaptic damage in brain tissue of CUMS mice via GATA1/ADRB2 regulation.**

Note: (A) H&E staining showing pathological changes in the hippocampal CA1, CA3, and DG regions of mice from each group (scale bar = 50 μm); (B) Nissl staining showing pathological changes in the hippocampal region of mice from each group (scale bar = 50 μm); black arrows indicate neuronal cell shrinkage and deformation; red boxes highlight extensive neuronal shrinkage and deformation in the hippocampus; (C) Representative IF images of Iba-1 (Red) and DAPI (Blue) merge in brain tissue (scale bar = 25 μm); (D) Quantification of Iba-1+ positive cells (normalized to control) in the prefrontal cortex (PFC); (E) IF staining for GFAP-positive astrocytes in brain tissue (scale bar = 25 μm); (F) Quantification of GFAP-positive astrocytes; (G) Representative images of TUNEL staining (Green), DAPI (Blue), and Merged channels in brain tissue (scale bar = 50 μm); (H) Quantification of TUNEL positive cells in each group. Data are presented as mean ± SEM. **p* < 0.05, ***p* < 0.01, ****p* < 0.001, *****p* < 0.0001. N = 6.

**Figure S9. Effects of Apt-EVs@GP on synaptic damage in CUMS mice via GATA1/ADRB2 regulation.**

Note: (A) Representative electron microscopy images showing the length, thickness of postsynaptic density (PSD), and synaptic gap width in the hippocampal CA1 and CA3 regions for each group (Ci and Cii: scale bar = 500 μm; ci and cii: scale bar = 200 μm); (B) PSD length in the CA1 region of the hippocampus for each group of mice; (C) PSD thickness in the CA1 region of the hippocampus for each group of mice; (D) Synaptic gap width in the CA1 region of the hippocampus for each group of mice; (E) PSD length in the CA3 region of the hippocampus for each group of mice; (F) PSD thickness in the CA3 region of the hippocampus for each group of mice; (G) Synaptic gap width in the CA3 region of the hippocampus for each group of mice. Data are presented as mean ± SEM. **p* < 0.05, ***p* < 0.01, ****p* < 0.001, *****p* < 0.0001. N = 6.

**Table S1. RT-qPCR primer sequences.**
